# Characterization of genome-wide segmental duplications reveals a common genomic feature of association with immunity among domestic animals

**DOI:** 10.1186/s12864-017-3690-x

**Published:** 2017-04-12

**Authors:** Xiaotian Feng, Jicai Jiang, Abinash Padhi, Chao Ning, Jinluan Fu, Aiguo Wang, Raphael Mrode, Jian-Feng Liu

**Affiliations:** 1 National Engineering Laboratory for Animal Breeding, Key Laboratory of Animal Genetics, Breeding and Reproduction, Ministry of Agriculture, College of Animal Science and Technology, China Agricultural University, Beijing, 100193 China; 2 Department of Animal and Avian Sciences, University of Maryland, College Park, MD 20740 USA; 3 International Livestock Research Institute, Nairobi, Box 30709–00100, Kenya

**Keywords:** Domestic animals, Segmental duplications, Copy number, Duplicated genes

## Abstract

**Background:**

Segmental duplications (SDs) commonly exist in plant and animal genomes, playing crucial roles in genomic rearrangement, gene innovation and the formation of copy number variants. However, they have received little attention in most livestock species.

**Results:**

Aiming at characterizing SDs across the genomes of diverse livestock species, we mapped genome-wide SDs of horse, rabbit, goat, sheep and chicken, and also enhanced the existing SD maps of cattle and pig genomes based on the most updated genome assemblies. We adopted two different detection strategies, whole genome analysis comparison and whole genome shotgun sequence detection, to pursue more convincing findings. Accordingly we identified SDs for each species with the length of from 21.7 Mb to 164.1 Mb, and 807 to 4,560 genes were harboured within the SD regions across different species. More interestingly, many of these SD-related genes were involved in the process of immunity and response to external stimuli. We also found the existence of 59 common genes within SD regions in all studied species except goat. These common genes mainly consisted of both UDP glucuronosyltransferase and Interferon alpha families, implying the connection between SDs and the evolution of these gene families.

**Conclusions:**

Our findings provide insights into livestock genome evolution and offer rich genomic sources for livestock genomic research.

**Electronic supplementary material:**

The online version of this article (doi:10.1186/s12864-017-3690-x) contains supplementary material, which is available to authorized users.

## Background

Repetitive DNA sequences are ubiquitous and these duplicated sequences occupy almost half of the human genome [1]. One type of DNA sequences among various repetitive sequences, with high sequence similarity (≥90%) and longer than 1kb, is called segmental duplication (SD). SDs tend to cluster within subtelomeric and pericentromeric regions, and the high similarity of SDs can lead to genomic rearrangement and recombination [2–5]. SDs are associated with non-allelic homologous recombination (NAHR) which may facilitate the formation of copy number variations (CNVs) [6–8]. SDs have been considered to play an important role in gene innovation, where genes embedded show a significant enrichment of biological functions in immunity, growth and responses to external stimuli [1, 9–12]. Recently, functional studies have unravelled that genetic diseases like Williams–Beuren syndrome and infertility are associated with genomic rearrangement caused by SDs on chromosomes 7 and Y, respectively, in the human genome [13, 14].

With the progress of sequencing projects moving forward, it is possible to explore the distribution, features and potential roles of duplicated sequences in genome evolution. Since the pioneer studies on SDs in human genome, several studies have been performed aiming at identification and characterisation of genome-wide SDs among other mammalian species such as mouse [9], rat [10], chimpanzee [11] and dog [12].

Although SDs are considered as one of the most important structural features in mammalian genomes, they have received little attention in most livestock species. So far, SDs have been merely systematically investigated in the genomes of bovine and swine [15, 16]. Liu et al. [16] reported a SD map of the bovine genome based on the version of bovine reference genome Btau 4.0.. Recently, we have constructed a SD map of the porcine genome based on the reference genome of Sscrofa10.2 [17], but the unmapped scaffolds have been largely ignored for SD detection therein.

For most of other livestock species, *i.e.*, horse, sheep, goat, rabbit and chicken, *etc*., seldom studies have been performed in-depth for SD characterization. Aiming at enhancing the understanding of the roles of SDs in genomic innovation and functional characterization of SDs across different species, we conducted global identification and comparison of SDs across seven livestock species in the current study. We applied two commonly used methods, *i.e.*, whole-genome assembly comparison (WGAC) and whole-genome shotgun sequence detection (WSSD) [3, 18] to explore genome-wide SDs in the genome of each species investigated. Our objectives herein lie in two aspects. Firstly, we present comprehensive SD profiles and comparison across the genomes of various livestock species, which will be beneficial to relevant studies on structural and functional genomics as well as evolutionary genetics related to SD regions; Secondly, we characterized and annotated SD regions across different species’ genomes to provide global insights into genomic structural features, further exploring potential functional genes and common mechanisms corresponding to SD regions.

## Methods

### Genome resources of domestic animals

All genomic data for SD analyses are from publicly-accessible databases. Genome assemblies for pig (Sscrofa10.2) [19], cattle (UMD3.1) [20], horse (EquCab2.0) [21], rabbit (OryCun2.0) [22], sheep (Oar_v3.1) [23] and chicken (Gallus_gallus-4.0) [24] were downloaded from Ensembl databases (ftp://ftp.ensembl.org/pub/), and those of cattle (Btau 4.6.1) [25] and goat (CHIR_1.0) [26] were downloaded from the NCBI FTP site (ftp://ftp.ncbi.nlm.nih.gov/genomes/). Meanwhile, we downloaded next generation sequencing (NGS) data of the individual of the reference genome for each species, *i.e.*, NGS data of porcine from the DDBJ FTP site (ftp://ftp.ddbj.nig.ac.jp/ddbj_database/dra/fastq/ERA009/ERA009086/), ovine and caprine from the NCBI FTP site (ftp://ftp-trace.ncbi.nlm.nih.gov/sra). Whole genome shotgun sequencing (WGS) sequence data of cattle, horse, rabbit and chicken were also downloaded from the NCBI FTP site (ftp://ftp-trace.ncbi.nlm.nih.gov/sra), which were then spliced to 36bp to simulate NGS data for WSSD analyses [27]. The resources of gene families were downloaded from HGNC database (HUGO Gene Nomenclature Committee, http://www.genenames.org/genefamilies/a-z).

### Segmental duplication detection

We used two different approaches to detect SDs in the genomes of seven domestic species, *i.e.*, WGAC and WSSD methods. All the details to implement the two approaches were illustrated in our previous study [17].

After finishing both WGAC and WSSD analyses for the reference genome, to further remove artifactual duplications, we filtered the WGAC alignments of ≥94% identity using the WSSD dataset. Following previous studies [9, 10, 12, 16, 18], the final SD database consisted of the combined results from the WGAC approach with identity <94% and the rest part filtered using the results of the WSSD approach (all custom Perl scripts are available at https://github.com/jiang18/sd_analysis). Finally, we constructed SD maps of domestic animals using the program Parasight v7.6 (http://eichlerlab.gs.washington.edu/jeff/parasight/index.html).

### Analyses of gene content within SD regions

We retrieved gene contents within SD regions based on genome annotation files downloaded from NCBI (*e.g.*, ftp://ftp.ncbi.nih.gov/genomes/Sus_scrofa/mapview/seq_gene.md.gz). Bioconductor (http://www.bioconductor.org/) was used to perform Gene Ontology (GO) analyses. Kyoto Encyclopedia of Genes and Genomes (KEGG) pathway analyses were conducted with DAVID (http://david.abcc.ncifcrf.gov/). Since only a limited number of genes in the livestock genomes have been annotated, we firstly converted the gene IDs of investigated livestock species to orthologous human Ensembl gene IDs by BioMart (http://www.biomart.org/), then carried out the GO and KEGG pathway analyses. We also analyzed orthologous protein-coding genes within SD regions among domestic animals based on OrthoDB release 7 (http://cegg.unige.ch/orthodb7). The phylogenetic trees were drawn using Clustal X (http://www.clustal.org/clustal2/) and Tree View (http://taxonomy.zoology.gla.ac.uk/rod/treeview.html).

### Association with other genomic landscapes

To further characterize identified SDs, we performed simulations to probe whether the identified SDs are associated with other genomic features, like CNVR, subtelomeric and pericentromeric regions and gene family regions. The simulation analyses were done by our self-developed Perl scripts. To test for association between SDs and CNVRs, we randomly assigned each of identified SD regions a putative position with no overlap with each other in the genome. The number or the length of CNVRs overlapping with SDs was calculated in each simulation, and finally, we created empirical distributions of the hits via 10,000 independent replications. Thus the significance of SD enrichment in CNV regions could be determined by the thresholds based on the empirical distributions. Similarly, associations of SDs with subtelomeric and pericentromeric regions as well as gene family regions were performed based on the same strategy. For the enrichment analyses, we defined approximate lengths of both subtelomeric and pericentromeric regions as 2 Mb based on previous studies of karyotype of each species [16, 28–36]. Considering the differences between avian genome and mammalian genome, subtelomeric and pericentromeric regions of several chromosomes in chicken genome were shortened to 300kb.

## Results

### Identification of segmental duplications

We identified segmental duplications among domestic animals based on two different approaches. Whole-genome assembly comparison (WGAC) is a BLAST-based approach to identify alignments with length ≥1kb and identity ≥90% [3], while whole-genome shotgun sequence detection (WSSD) can find SD regions by calculating mapping read depth [18, 37]. After removing “artifactual duplications”, we identified the SD regions among domestic animals by combining the filtered results of WGAC approach and the results of WSSD approach.

For WGAC analyses, the initial results were significantly different among the seven species investigated, ranging from 54,933 pairwise alignments (goat) to 902,537 pairwise alignments (pig). After removing high-copy repeats, the number of pairwise alignments for most of the investigated species reduced to ~20,000 and the rabbit genome had the largest amount of alignments, with 54768 (Table 1). The number of alignments decreased in porcine genome dramatically, which may be due to the filtration of initial alignments of high similarity. According to previous studies, SDs showed a significant enrichment in unassigned scaffolds [3, 12, 16]. Compared with other 6 species, rabbit genome has larger number of unassigned scaffolds (17.9%, 489.7 Mb of 2,737.4Mb), which may account for its larger number of pairwise alignments.

**Table 1:** The amount of initial and cleaned pairwise alignments for each of 7 domestic species investigated

Species	Initial alignments	Cleaned alignments
Cattle	63,978	16,402
Pig	902,537	20,000
Horse	70,532	14,841
Rabbit	338,613	54,768
Goat	54,933	14,403
Sheep	107,362	11,719
Chicken	111,922	25,823

The initial WGAC pairwise alignments were filtered by custom Perl programs. Duplications with 50 or more copies within the genome or present on 3 or more chromosomes were removed, generating cleaned pairwise alignments

Specifically, we identified 31,148 pairs of alignments in the Btau 4.6 genome assembly for cattle, among which 18,872 (60.6%) involved unmapped scaffolds. In contrast, only 1,019 in 13,946 pairs of alignments involved unmapped alignments in the UMD 3.1 assembly. Btau 4.6 is the sole livestock genome assembly with the Y chromosome in our study. Surprisingly, 9,954 pairs of alignments (32.0%) involved the Y chromosome, among which 3793 pairs (38.1%) were linked to unmapped scaffolds. Since we were more interested in chromosomes than unmapped scaffolds, we focused on UMD 3.1 for further analyses of cattle genome.

**Fig. 1:**
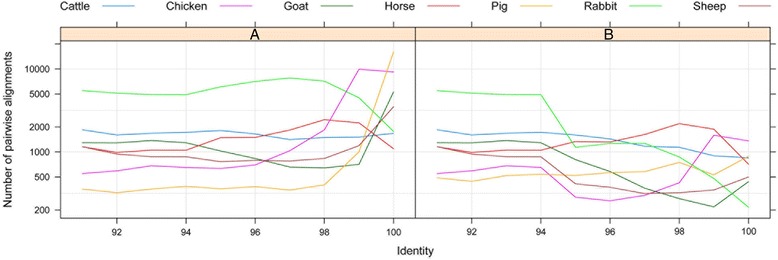
Identity distribution of pairwise alignments. Panel **a** shows the identity distribution of pairwise alignments based on the cleaned results of WGAC approach, while panel **b** displays the WGAC results filtered by WSSD approach. Each color represents one species

The identity distributions of alignments are showed in Fig. 1. The curve of alignments with identity from 90-96% largely keeps constant in most of the investigated species, while varying significantly out of this interval among different domestic species. Accordingly, in the identified interval of 96–100%, the distribution curves of porcine, ovine, caprine and chicken alignments with identity ≥94% need to be filtered with results of WSSD approach to remove “artifactual duplications”.

In WSSD analyses, there were 4,994, 924, 1,829, 1,226, 2,028, 1,959 and 948 SD intervals (with length ≥10kb) identified for cattle, pig, horse, rabbit, goat, sheep and chicken, respectively (Additional file 1: Table S1.1-7). Average absolute copy numbers of these intervals ranged from 6.7 (rabbit) to 12.0 (pig) and for each species were 9.1, 12.0, 10.1, 6.7, 11.6, 11.3 and 6.9, respectively.

After removing “artifactual duplications”, we finally determined the SD contents of seven domestic species. For bovine, porcine, equine, rabbit, caprine, ovine and chicken genome, the SD contents of the genome were 2.6% (68.2 Mb of 2,670.4 Mb), 2.0% (57.3 Mb of 2,808.5 Mb), 6.6% (164.1 Mb of 2,474.9 Mb), 5.1% (139.7 Mb of 2,737.5 Mb), 3.4% (90.2 Mb of 2,635.8 Mb), 3.3% (87.0 Mb of 2,619.0 Mb) and 2.0% (21.7 Mb of 1,100.5 Mb), respectively (Additional file 2: Table S2, Additional file 3: Table S3.1-7). These contents were similar to other mammalian species studied before, like dog [12] and human [18]. The chicken genome with the smallest reference genome had the lowest content. We conjectured that SD content depends on the scale of reference genome and the unmapped scaffolds. Finally, we constructed SD maps of seven domestic species (Additional file 4: Figure S1.1-7).

We specifically investigated the proportion of WGAC detected long SDs (>10 kb, >94% similarity) verified by the WSSD results (Table 2). A low proportion implied that the genome assembly had a more serious issue in distinguishing SDs.

**Table 2:** Copy numbers of genes in SD regions for domestic species

Species	Genome assembly	Length of WGAC hits >10kb	Length of WGAC hits >10kb filtered by WSSD	Proportion of filtered WGAC hits
Cattle	Btau 4.6 (excluding Y)	69,558,932	51,891,155	0.746
Cattle	UMD 3.1	19,740,894	18,397,219	0.932
Pig	Sscrofa 10.2	23,482,697	20,311,340	0.865
Horse	EquCab 2.0	59,554,690	56,165,607	0.943
Rabbit	OryCun 2.0	24,688,548	16,130,349	0.653
Goat	CHIR 1.0	2,093,238	1,946,680	0.930
Sheep	Oar_v3.1	1,044,992	888,894	0.851
Chicken	Ggallus 4.0	3,020,167	2,268,769	0.751

### Distribution of segmental duplications

SD regions were dispersed across the genome for each of the investigated species. We calculated total length of SDs on each chromosome for seven domestic species (Additional file 4: Figure S2.1-7, Figure S3.1-7).

Interestingly, SD regions for most investigated species (5 out of 7 species, including cattle, pig, horse, goat and sheep) were overlong in the X chromosome, especially for cattle and goat. Notably, in chicken genome, chromosome 26 had no pairwise alignments detected by WGAC approach, and no duplicated region with length ≥10 kb identified by WSSD approach as well. Due to the poor annotation of chicken genome [38], no SDs in chromosome W was identified by both two approaches (only 10 short segments were detected in W_Random chromosome).

For bovine, porcine, equine, rabbit and chicken genomes, intrachromosomal duplications were much more than interchromosomal duplications excluding unmapped scaffolds. For porcine, equine and chicken genome, interchromosomal duplications had higher sequence identity than intrachromosomal duplications. Inversely in the caprine and rabbit genomes, the majority of alignments between chromosomes had a low sequence identity of ≤94%.

Previous studies revealed that SDs account for high proportion of contents on unmapped scaffolds [1, 9–12, 16, 39]. Except porcine genome, over 10% of unmapped scaffolds were identified as SD regions and the proportion reaches 40% for equine genome (44.1 out of 107.9 MB). The enrichment of SDs in unmapped scaffolds in these domestic species was similar to previous studies and the high identity of SDs became a tremendous obstacle encountered when we mapped these segments to reference genome.

Similar to human, mouse and dog genomes [1, 9, 12], SDs were enriched in subtelomeric and pericentromeric regions among seven domestic species. Because of the imprecise determination of telomeric and centromeric regions of domestic species, we considered approximate subtelomeric and pericentromeric regions based on previous studies [28–31, 34, 36, 40]. SDs of these seven domestic species showed significant enrichment in pericentromeric regions, *i.e.*, 5.5-fold (*P* < 0.0001) for bovine genome, 4.8-fold (*P* < 0.0001) for porcine genome, 8.7-fold (*P* < 0.0001) for equine genome, 1.8-fold (*P* < 0.0001) for rabbit genome, 9.3-fold (*P* < 0.0001) for caprine genome, 3.8-fold (*P* < 0.0001) for ovine genome and 3.5-fold (*P* < 0.0001) for chicken genome. For subtelomeric regions, SDs were enriched with 1.8-fold (*P* < 0.0001), 16.4-fold (*P* < 0.0001), 3.6-fold (*P* < 0.0001), 2.8-fold (*P* < 0.0001), 2.7-fold (*P* < 0.0001), 1.8-fold (*P* < 0.0001) and 2.3-fold (*P* < 0.0001) for cattle, pig, horse, rabbit, goat, sheep and chicken, respectively. This indicated that the enrichment of SDs in subtelomeric and pericentromeric regions occurred in majority of domestic species.

The repeat properties of SD regions among domestic species were summarized in Additional file 5: Table S4. The content of each repeat category was similar with each other among six mammalian species, while an obviously different feature existed in the chicken genome in contrast to other six mammalian species. Specifically, the DNA elements of SDs in chicken genome was slightly less than mammalian genome, while the average length of SDs in chicken genome was nearly twice longer than that of SDs in mammalian genomes; For long interspersed elements (LINEs) and short interspersed elements (SINEs), both the number and the average length of the avian genome was extremely lower than those of mammalian species.

### Gene content of segmental duplications

Based on the gene information of each species from NCBI, we found 3,734, 3,096, 3,690, 2,924, 2,460, 4,560 and 807 genes in SD regions identified in bovine, porcine, equine, rabbit, caprine, ovine and chicken genomes, respectively. We calculated the copy numbers of those genes. Average copies of genes ranged from 4.8 to 11.9 (11.9 for bovine genome, 7.3 for porcine genome, 5.5 for equine genome, 4.8 for rabbit genome, 4.9 for caprine genome, 5.5 for ovine genome and 6.6 for chicken genome). Half of genes had more than two copies, mainly ranging from 3 to10 copies (Table 3).

**Table 3:** Proportion of WGAC-detected SDs verified by WSSD

	Species						
CN	Cattle	Pig	Horse	Rabbit	Goat	Sheep	Chicken
≤1.5	39	19	29	21	4	6	7
1.5–2.5	644	712	604	694	924	1146	149
2.5–10.5	1049	720	1522	1000	670	927	463
10.5–20.5	300	66	134	114	34	64	32
20.5–30.5	72	14	29	22	2	5	20
30.5–40.5	26	1	15	5	3	2	3
40.5–50.5	15	2	5	3	1	2	4
50.5–60.5	7	0	4	2	1	0	3
60.5–70.5	6	1	0	3	2	1	1
70.5–80.5	4	1	1	0	3	0	2
80.5–90.5	4	1	1	0	0	0	2
90.5–100.5	1	0	0	0	0	2	2
≥100.5	41	13	5	1	3	9	1
Null	1527	1547	1340	1059	814	2396	119
Total	3735	3097	3689	2924	2461	4560	808
Ave.CN	11.9	7.3	5.5	4.8	4.9	5.5	6.6

To in-depth exploit potential functions of genes within SD regions among various species, we performed Gene Otology (GO) and KEGG pathway enrichment analyses on all genes within SD regions for each species surveyed. Overall, similar to the results of previous studies in human [18], mouse [9], rat [10], chimpanzee [11], dog [12] and silkworm [39], we found that genes in SD regions were largely enriched with functions and process of immunity, growth and responses to external stimuli for most of these mammalian species.

Specially, for GO terms, we found that genes in SD regions of five species (dog, cattle, pig, horse and sheep) were commonly enriched in xenobiotic metabolic process and response to xenobiotic stimuli (Additional file 6: Table S5.1). For molecular function ontology, genes of most species (8 out of 10 species, except goat and chicken) were enriched in glucuronosyltransferase activity which is related to drug metabolism (Additional file 6: Table S5.2) [41]. Different from mammalian species, genes in SD regions of the chicken genome were mainly enriched in cell projection organization and neuron projection development. This may due to the differences of evolution course between chicken and mammalian species. In pathway enrichment analyses, those significant pathway-enriched genes in most species were mainly associated with detoxification and metabolism process (Additional file 7: Table S6). It is notable that the olfactory transduction pathway contains the largest amount of olfactory receptor genes in bovine, porcine, equine and rabbit genomes. These olfactory receptor proteins have been reported as one of the main duplicated gene families [42–44].

**Fig. 2:**
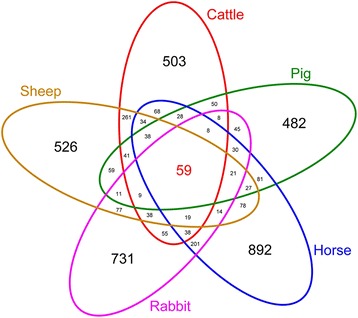
Common genes among 5 mammalian domestic species. The Venn diagram shows the number of common genes among 5 mammalian domestic species. Each color represents one species

**Fig. 3:**
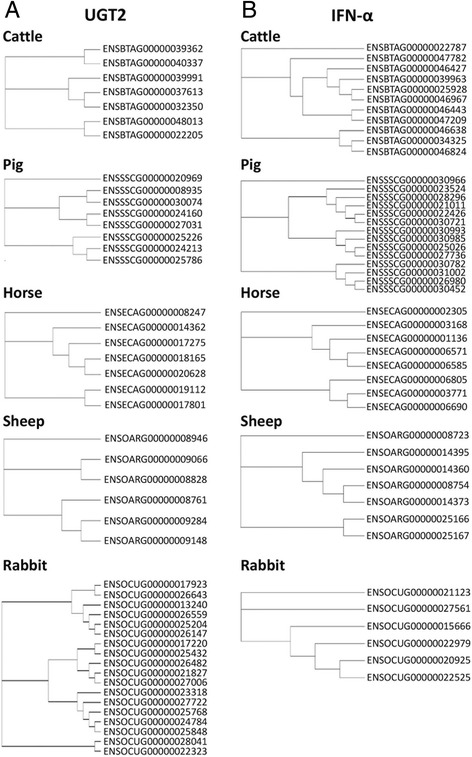
Phylogenetic trees of UGT2 and IFNA gene families of 5 domestic species. Phylogenetic trees of UGT2 gene family are displayed in panel A, while the results of IFN-α gene family are showed in panel B. Only tandem cluster of genes in each family are considered. The phylogenetic trees were constructed using ClustalX (http://www.clustal.org/clustal2/) and Tree View (http://taxonomy.zoology.gla.ac.uk/rod/treeview.html)

To seek the exact genes commonly embedded in SD regions among different species, we converted IDs of genes of livestock species to human homologous gene IDs for further comparison. We picked out a total number of 304 common genes within SD regions of at least five species (listed in Additional file 8: Table S7). We then investigated whether these 304 common genes were enriched in certain pathways and involved in some common biology processes (Table 4). Accordingly, we found that these common genes played a crucial role in the enrichment of immunity and response to external stimuli. Considering the relatively poor gene annotation in caprine genome as well as the specialization of chicken genome, we finally determined 59 genes as mutual genes in SD regions among domestic species including cattle, pig, horse, rabbit and sheep (Fig. 2, Additional file 9: Table S8). These 59 SD-harbored common genes mainly belong to four gene families, *i.e.*, UDP glucuronosyltransferases (UGTs), interferons (IFNs), histones and olfactory receptors (ORs). Intriguingly, both of UGTs and IFN gene families are significantly enriched in SD regions (*P* < 0.0001) across the genomes of all livestock species. The phylogenetic trees of detected genes of UGT2 and IFN-α families within SD regions for 5 mammalian species were showed in Fig. 3. Previous reports have shown that UGTs transfer the glucuronic acid component of UDP-glucuronic acid to a small hydrophobic molecule which is associated with xenobiotic metabolic process in liver [45], and IFNs are the proteins for defencing external viruses which is made and released by host cells [46]. This provides an important evidence on the potential roles of SDs associated with immunity and responses to external stimuli due to the functions of these two gene families being widely present in the SD regions across the genomes of majority of mammalian species.

**Table 4:** KEGG pathway enrichment analysis of common genes in SD regions among domestic species

KEGG Term	Description	Count	*P-*Value	Fold Enrichment	Bonferroni
hsa00980	Metabolism of xenobiotics by cytochrome P450	22	8.33E-20	14.92	7.00E-18
hsa00982	Drug metabolism	22	1.84E-19	14.43	1.55E-17
hsa00830	Retinol metabolism	20	4.83E-18	15.07	4.05E-16
hsa00983	Drug metabolism	16	2.32E-14	15.14	1.95E-12
hsa00140	Steroid hormone biosynthesis	16	7.37E-14	14.15	6.18E-12
hsa05320	Autoimmune thyroid disease	15	7.57E-12	11.96	6.36E-10
hsa04740	Olfactory transduction	35	7.62E-12	3.76	6.40E-10
hsa04140	Regulation of autophagy	13	1.34E-11	15.11	1.13E-09
hsa04623	Cytosolic DNA-sensing pathway	14	3.49E-10	10.35	2.93E-08
hsa04612	Antigen processing and presentation	16	8.22E-10	7.84	6.90E-08
hsa04622	RIG-I-like receptor signaling pathway	14	1.01E-08	8.02	8.45E-07
hsa00053	Ascorbate and aldarate metabolism	8	6.88E-08	19.14	5.78E-06
hsa00040	Pentose and glucuronate interconversions	8	1.10E-07	18.08	9.26E-06
hsa04620	Toll-like receptor signaling pathway	14	7.61E-07	5.64	6.39E-05
hsa00150	Androgen and estrogen metabolism	9	2.13E-06	9.90	1.79E-04
hsa00500	Starch and sucrose metabolism	9	5.87E-06	8.72	4.93E-04
hsa00860	Porphyrin and chlorophyll metabolism	8	1.09E-05	9.86	0.000916
hsa04650	Natural killer cell mediated cytotoxicity	14	1.72E-05	4.28	0.001442
hsa00591	Linoleic acid metabolism	7	4.52E-05	10.17	0.003790
hsa04630	Jak-STAT signaling pathway	14	8.71E-05	3.67	0.007294

### Association of SDs with gene families

It has been reported that gene duplication and conversion are important sources of the evolution of gene families, including those with uniform members and those with diverse functions [47]. To explore association between SDs and various gene families, we further investigated potential enrichment of gene families in SD regions. We firstly collected the gene families from human genome HGNC database and mapped them to the corresponding livestock genome investigated according to the orthology between human and each of species. We then tested the enrichment of gene families in the corresponding genome via simulation based on two different criteria, *i.e.*, the length of genes overlapping with SD regions as well as the number of genes involved in SD regions. As shown in Table 5, we found that gene families were enriched in SD regions (*P < 0.001*) in contrast to non-family genes among common domestic species.

**Table 5:** Enrichment fold of gene families in SD regions in 7 domestic genomes

	Species						
	Cattle	Pig	Horse	Rabbit	Goat	Sheep	Chicken
Length	1.40	4.99	2.97	2.72	1.26	2.61	2.57
Number	1.57	4.07	2.63	2.03	1.88	2.38	1.08

To test the enrichment of gene families in SD regions, we considered the total length and the total number of genes from all gene families in each simulation, respectively. Significant enrichment was found in all 7 domestic genomes investigated (*P < 0.0001*)

### Gene orthology within SD regions

To survey common features of SDs across various livestock species, we sifted out a total number of 89 orthologous genes within SD regions of all livestock species according to the resources of OrthoDB [48] (Additional file 10: Table S9). Surprisingly, we found orthologous genes in SD regions also showed enrichment of immune response, olfactory receptor activity, G-protein coupled receptor activity and sensory perception of smell. Furthermore, we found that the orthology group EOG6R518B commonly presented among all nine species except pig, which were mainly associated with functions of carboxypeptidase activity and signal transduction.

## Discussion

To our knowledge, this is the first global analysis of segmental duplications among a majority of domestic animals. We identified genome-wide SDs in bovine, porcine, equine, rabbit, caprine, ovine and chicken genomes. The distribution and features of SDs in mammalian domestic species were similar to previous studies in rat and mouse, while SDs in the chicken genome had obviously different characteristics. Fifty-nine common genes were identified in SD regions across five mammalian domestic species and showed significant enrichment in immunity function and responses to external stimuli. Our studies presented valuable resources for further systematic investigation of duplicate blocks, duplicate genes and CNV formation. This will benefit the genome assemblies of domestic species with better understanding of these duplicated sequences on unmapped scaffolds as well. It is notable that the SDs detected were based on the reference genomes released before the beginning time of current study. It should be preferable to employ the latest version of the reference genome to update the SD database herein in our future endeavours.

As we all known, segmental duplications are long DNA sequences (typically defined as being > 1kb in length) that have nearly identical sequences (90-100%) and exist in multiple locations as a result of duplication events. However, there are three possible outcomes when large nearly identical duplicated sequences are encountered during sequence and assembly: (1) The sequences may be recognized as distinct and properly resolved as separate loci, (2) the sequences may be underrepresented due to the presence of virtually identical sequence already in the database, or (3) distinct paralogous loci may be mistakingly assembled into a single sequence contig [4]. Example, In the SD study of human, It had been discussed the likelihood that highly similar (for example, >98% identity) apparent intrachromosomal duplications may be erroneous [18, 49]. Meanwhile, It realized that many duplicated regions in current, published genome sequences are in fact errors due to mis-assembly [50]. Therefore, the complete genome were more prior to correct the false segmental duplications caused by genome mis-assembly and detect more accurate segmental duplications.

Chicken is the first sequenced domestic species and is a crucial avian livestock in many countries [51]. However, unmapped scaffolds still took up 4.0% of the chicken genome. According to our study, over 1/10 (7.2 Mb of 68.6 Mb) of these unmapped sequences consisted of segmental duplications. These high-identity sequences are obstacles for genome assembly. The chicken genome showed different SD features from mammalian domestic species. No SDs in chromosome W were identified in our study. This may be due to the limited genetic diversity of chromosome W which is influenced by sex-linked selection [52]. Totally different from mammalian species, genes in SD regions in the chicken genome showed enrichment in cell projection organization and neuron projection development which shared no similar function with those in mammalian species.

In our study, we found that all the investigated mammalian livestock showed enrichment of SDs in subtelomeric and pericentromeric regions. Besides, genes harboured in SD regions were enriched in immunity functions and responses to external stimuli in most of the mammalian animals.

Based on our results, over half of genes in SD regions have multi-copies ranging from 4.8 to 11.9. We found 11 genes with more than 5 copies among all of our investigated domestic animals as well as in human, mouse and dog genome. Interestingly, most of these multi-copy genes were pseudo genes and were associated with sex-related functions. In bovine genome, a tandem cluster of pseudo genes on chromosome 17 were found in SD regions, which were associated with testis-specific Y-encoded protein. According to previous studies, testis specific protein Y-encoded (TSPY) was a tandem cluster of genes with multi-copies ranged from 50–200 copies in cattle genome [53, 54]. Zinc finger (ZNF) genes were found in all domestic species. This gene family was also reported as tandem gene clusters in mammalian genomes [55, 56]. In human genome [57], ZNF gene clusters were located in pericentromeric region of chromosome 10 and with divergence caused by inversion events. This also provided an evidence for the genomic rearrangement facilitated by segmental duplications. In addition, genes with more than 100 copies which encode spermatogenesis-associated protein were discovered in SD regions of equine genome. Prostaglandin D2 synthase 21kDa (brain) (PTGDS) from chicken genome had copy numbers near 100 copies, which was associated with a male-specific pathway as well [58]. Previous studies revealed that this type of multigene family consists of genes derived from duplication, deletion and inversion events of a common ancestral gene [55–57]. Based on our results, we suspected that segmental duplications with high identity could facilitate the occurrence of duplication, deletion and inversion events, further leading to more complex gene variation.

In the current study, 59 common genes were found in SD regions among five mammalian domestic species. These genes mainly consisted of four gene families, *i.e.*, UGTs, IFNs, histones and ORs. UGT gene superfamily of mammalian species could be divided into four families, UGT1, UGT2, UGT3 and UGT8 [59]. All members of UGT2B family were included in these 59 common genes and the copy numbers ranged from 4–6 among different species. A previous study showed that genes in this family were closely linked among different species, but there was no evidence to prove that these genes were truly orthologous [60]. Furthermore, UGT2B17 was the most attractive one of UGT2B family and had been extensively studied previously. Polymorphic deletions were detected in UGT2B17 and UGT2B28 and segmental duplications were found near these genes [61, 62], which were associated with osteoporosis risk related to the occurrence of NAHR caused by segmental duplications [63, 64]. Thus, we suggested that the high identity and polymorphism of UGT2B gene family were strongly connected with the genomic rearrangement occurred by segmental duplications. Besides, all members of IFN alpha (IFN-α) gene family were listed in the 59 common genes found in SD regions among 5 mammalian domestic species. Previous studies revealed that divergence of type I IFN was associated with rearrangements and the expansion of IFNA gene family was caused both by duplication and conversion events [65, 66]. In the current study, common genes in the identified SD regions in multiple genomes revealed their association with immunity and response to external stimuli, especially for detoxification and drug metabolism. This might be the representative and salient characteristic of genes in SD regions. In-depth comparative analyses of function and expression of these genes among different species need to be further explored.

## Conclusions

In summary, we conducted the first detailed and comparative analyses of SDs among major domestic animals to identify the SD content, characterize the feature of SDs, and annotate genes in SD regions of each species. The construction of SD maps of common domestic species offered abundant genomic resources for related studies in the future. Common genes with function of immunity and response to external stimuli were found in SD regions among the analysed mammalian domestic species. Our findings herein offer a valuable resource to facilitate both comparative genomic as well as structural genomic studies.

## Additional files

Additional file 1: Table S1.1-7. SD regions of 7 domestic species detected by WSSD method. (XLSX 772 kb)

Additional file 2: Table S2. The distribution of SDs among 7 domestic species. (XLSX 10 kb)

Additional file 3: Table S3.1-7. SD regions of 7 domestic species combining results of WGAC and WSSD. (XLSX 1285 kb)

Additional file 4: Supplemental Figure. Including **Figures S1–S3**. (PDF 2259 kb)

Additional file 5: Table S4. Repeat properties of SD regions among domestic species. (XLSX 12 kb)

Additional file 6: Table S5. GO analyses of genes detected in SD regions. (XLSX 15 kb)

Additional file 7: Table S6. KEGG pathway analyses of genes detected in SD regions. (XLSX 11 kb)

Additional file 8: Table S7. Human homologous genes detected in SD regions. (XLSX 29 kb)

Additional file 9: Table S8. Fifty-nine human homologous genes detected in SD regions among five domestic species. (XLSX 10 kb)

Additional file 10: Table S9. Analyses of genes detected in SD regions based on OrthoDB. (XLSX 13 kb)

## Abbreviations

CNVs: Copy number variations
GO: Gene Ontology
IFNs: Interferons
IFN-α: IFN alpha
KEGG: Kyoto Encyclopedia of Genes and Genomes
LINEs: Long interspersed elements
NAHR: Non-allelic homologous recombination
NGS: Next generation sequencing
ORs: Olfactory receptors
PTGDS: Prostaglandin D2 synthase
SDs: Segmental duplications
SINEs: Short interspersed elements
TSPY: Testis specific protein Y-encoded
UGTs: UDP glucuronosyltransferases
WGAC: Whole-genome assembly comparison
WGS: Whole genome shotgun sequencing
WSSD: Whole-genome shotgun sequence detection
ZNF: Zinc finger
