# Supplementary material for: Characterization of genome-wide segmental duplications reveals a common genomic feature of association with immunity among domestic animals
Source: BMC Genomics. 2017 Apr 12;18:293. doi: 10.1186/s12864-017-3690-x (PMC5389087; doi:10.1186/s12864-017-3690-x)
Supplement: Supplementary file 4 — Supplemental Figure. Including Figures S1–S3. (PDF 2259 kb) [file 12864_2017_3690_MOESM4_ESM.pdf]

Supplemental Figure

Figure S1. SD maps of domestic species

Figure S1.1 SD maps of bovine genome

Figure S1.2 SD maps of porcine genome

Figure S1.3 SD maps of equine genome

Figure S1.4 SD maps of rabbit genome

Figure S1.5 SD maps of caprine genome

Figure S1.6 SD maps of ovine genome

Figure S1.7 SD maps of chicken genome

Only SDs with length ≥5 kb are exhibited. Intrachromosomal pairwise alignments are collected by blue line, and the interchromosomals are marked with short red lines. The map was drawn using the program parasight v7.6 (http://eichlerlab.gs.washington.edu/jeff/parasight/index.html).

Figure S2. Total length of SD regions on each chromosome of 7 domestic species

Figure S2.1 Total length of SD regions on each chromosome of bovine genome

Figure S2.2 Total length of SD regions on each chromosome of porcine genome

Figure S2.3 Total length of SD regions on each chromosome of equine genome

Figure S2.4 Total length of SD regions on each chromosome of rabbit genome

Figure S2.5 Total length of SD regions on each chromosome of caprine genome

Figure S2.6 Total length of SD regions on each chromosome of ovine genome

Figure S2.7 Total length of SD regions on each chromosome of chicken genome

Chromosome with high content of SDs is marked with red. For better comparison of SD content between different species, we use the uniform coordinate axis among 7 species.

Figure S3. Distribution of SDs (length ≥5 kb) among 7 domestic species

Figure S3.1 Distribution of SDs (length ≥5 kb) of bovine genome

Figure S3.2 Distribution of SDs (length ≥5 kb) of porcine genome

Figure S3.3 Distribution of SDs (length ≥5 kb) of equine genome

Figure S3.4 Distribution of SDs (length ≥5 kb) of rabbit genome

Figure S3.5 Distribution of SDs (length ≥5 kb) of caprine genome

Figure S3.6 Distribution of SDs (length ≥5 kb) of ovine genome

Figure S3.7 Distribution of SDs (length ≥5 kb) of chicken genome

Only SDs with length ≥5 kb are exhibited. Pairwise alignments with identity＜94% are drawn in red lines, and pairwise alignments with identity ≥94% are marked in blue lines. The result was drawn using the program Circos (http://circos.ca/)

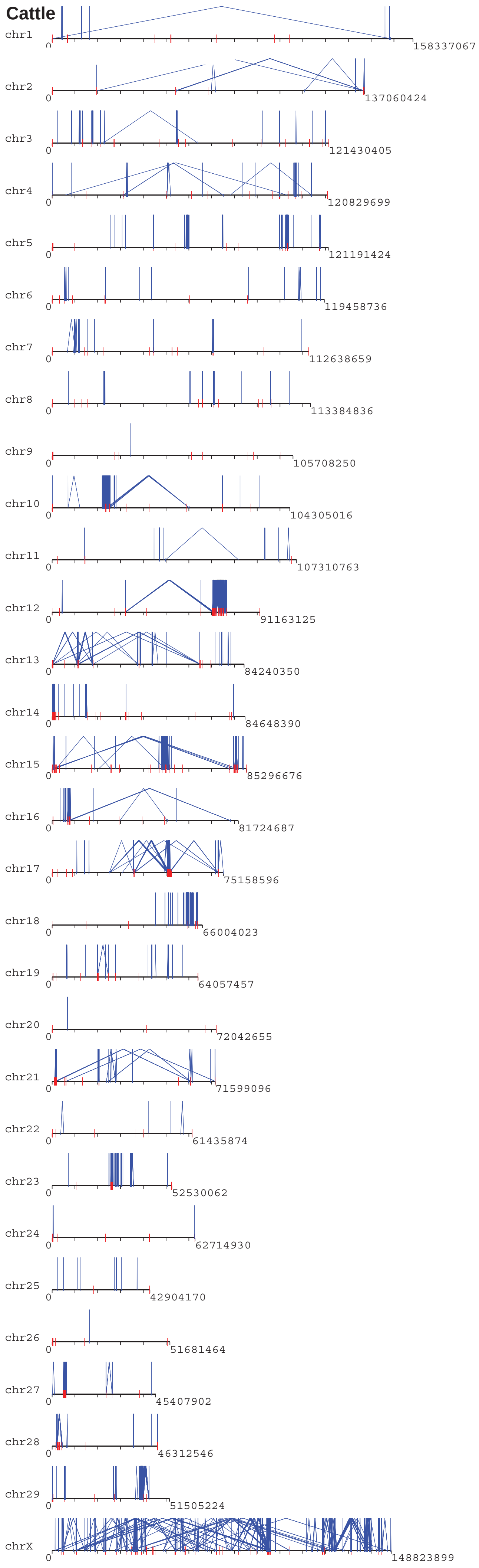
Cattle
chr1
0
158337067
chr2
0
137060424
chr3
0
121430405
chr4
0
120829699
chr5
0
121191424
chr6
0
119458736
chr7
0
112638659
chr8
0
113384836
chr9
0
105708250
chr10
0
104305016
chr11
0
107310763
chr12
0
91163125
chr13
0
84240350
chr14
0
84648390
chr15
0
85296676
chr16
0
81724687
chr17
0
75158596
chr18
0
66004023
chr19
0
64057457
chr20
0
72042655
chr21
0
71599096
chr22
0
61435874
chr23
0
52530062
chr24
0
62714930
chr25
0
42904170
chr26
0
51681464
chr27
0
45407902
chr28
0
46312546
chr29
0
51505224
chrX
0
148823899

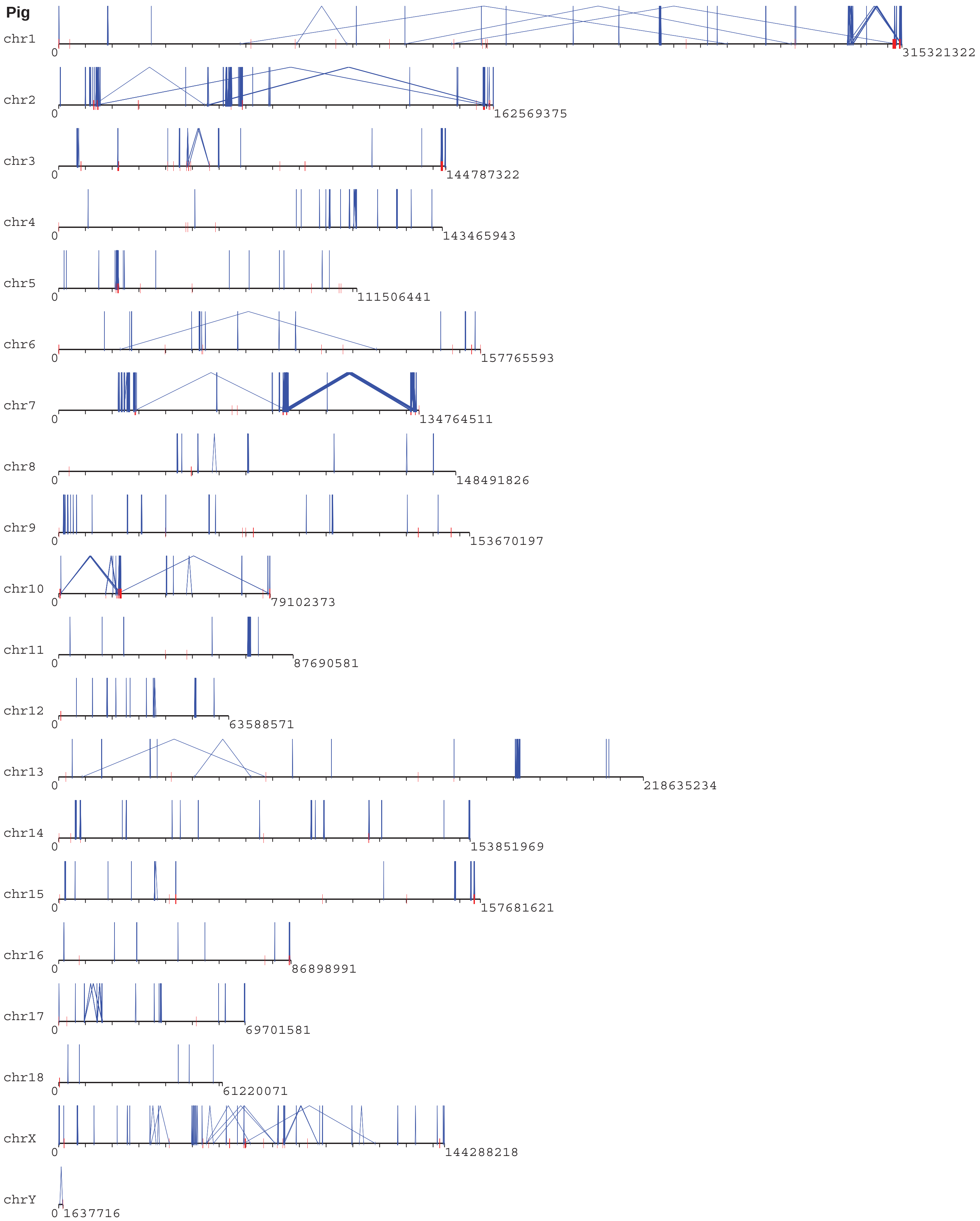
Pig
chr1
0
315321322
chr2
0
162569375
chr3
0
144787322
chr4
0
143465943
chr5
0
111506441
chr6
0
157765593
chr7
0
134764511
chr8
0
148491826
chr9
0
153670197
chr10
0
79102373
chr11
0
87690581
chr12
0
63588571
chr13
0
218635234
chr14
0
153851969
chr15
0
157681621
chr16
0
86898991
chr17
0
69701581
chr18
0
61220071
chrX
0
144288218
chrY
0
1637716

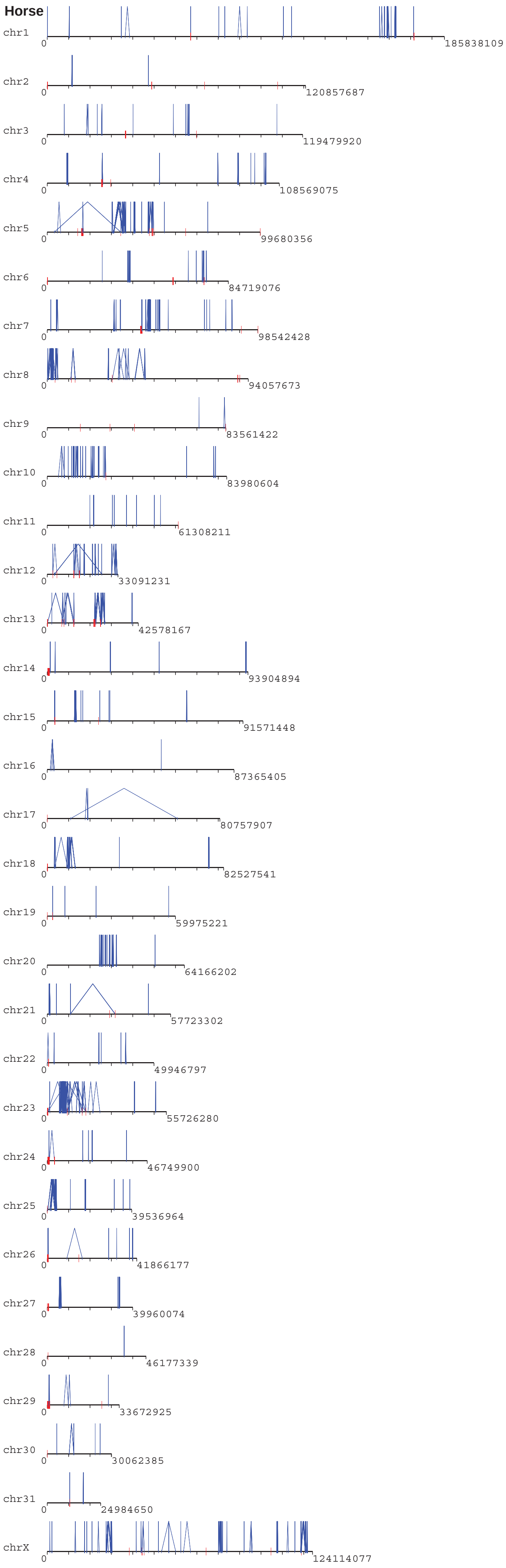
Horse
chr1
0
185838109
chr2
0
120857687
chr3
0
119479920
chr4
0
108569075
chr5
0
99680356
chr6
0
84719076
chr7
0
98542428
chr8
0
94057673
chr9
0
83561422
chr10
0
83980604
chr11
0
61308211
chr12
0
33091231
chr13
0
42578167
chr14
0
93904894
chr15
0
91571448
chr16
0
87365405
chr17
0
80757907
chr18
0
82527541
chr19
0
59975221
chr20
0
64166202
chr21
0
57723302
chr22
0
49946797
chr23
0
55726280
chr24
0
46749900
chr25
0
39536964
chr26
0
41866177
chr27
0
39960074
chr28
0
46177339
chr29
0
33672925
chr30
0
30062385
chr31
0
24984650
chrX
0
124114077

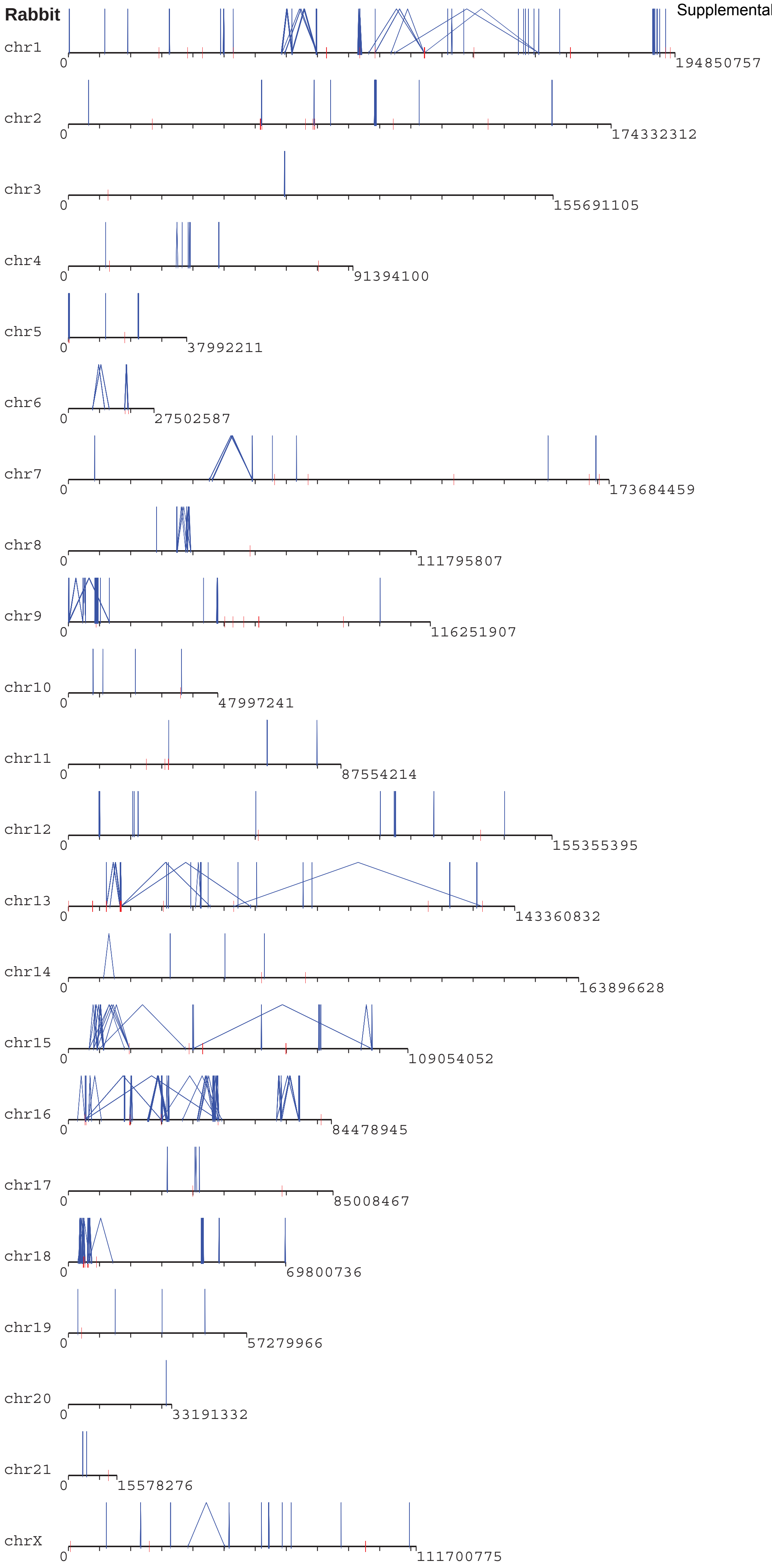
Rabbit
chr1
0
194850757
chr2
0
174332312
chr3
0
155691105
chr4
0
91394100
chr5
0
37992211
chr6
0
27502587
chr7
0
173684459
chr8
0
111795807
chr9
0
116251907
chr10
0
47997241
chr11
0
87554214
chr12
0
155355395
chr13
0
143360832
chr14
0
163896628
chr15
0
109054052
chr16
0
84478945
chr17
0
85008467
chr18
0
69800736
chr19
0
57279966
chr20
0
33191332
chr21
0
15578276
chrX
0
111700775

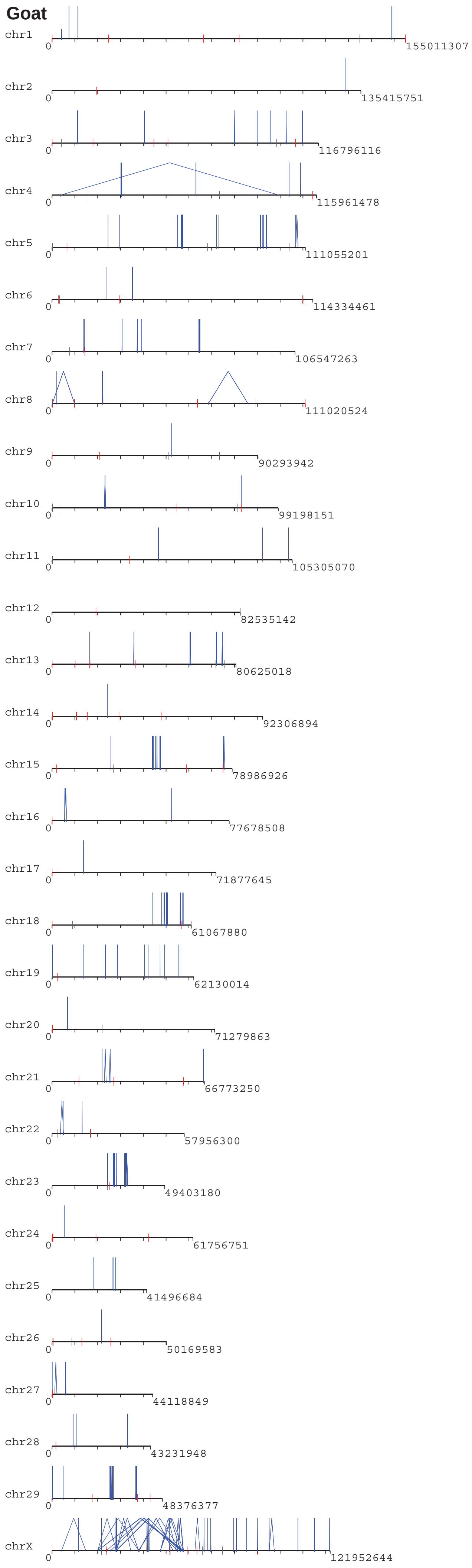
Goat
chr1
0
155011307
chr2
0
135415751
chr3
0
116796116
chr4
0
115961478
chr5
0
111055201
chr6
0
114334461
chr7
0
106547263
chr8
0
111020524
chr9
0
90293942
chr10
0
99198151
chr11
0
105305070
chr12
0
82535142
chr13
0
80625018
chr14
0
92306894
chr15
0
78986926
chr16
0
77678508
chr17
0
71877645
chr18
0
61067880
chr19
0
62130014
chr20
0
71279863
chr21
0
66773250
chr22
0
57956300
chr23
0
49403180
chr24
0
61756751
chr25
0
41496684
chr26
0
50169583
chr27
0
44118849
chr28
0
43231948
chr29
0
48376377
chrX
0
121952644

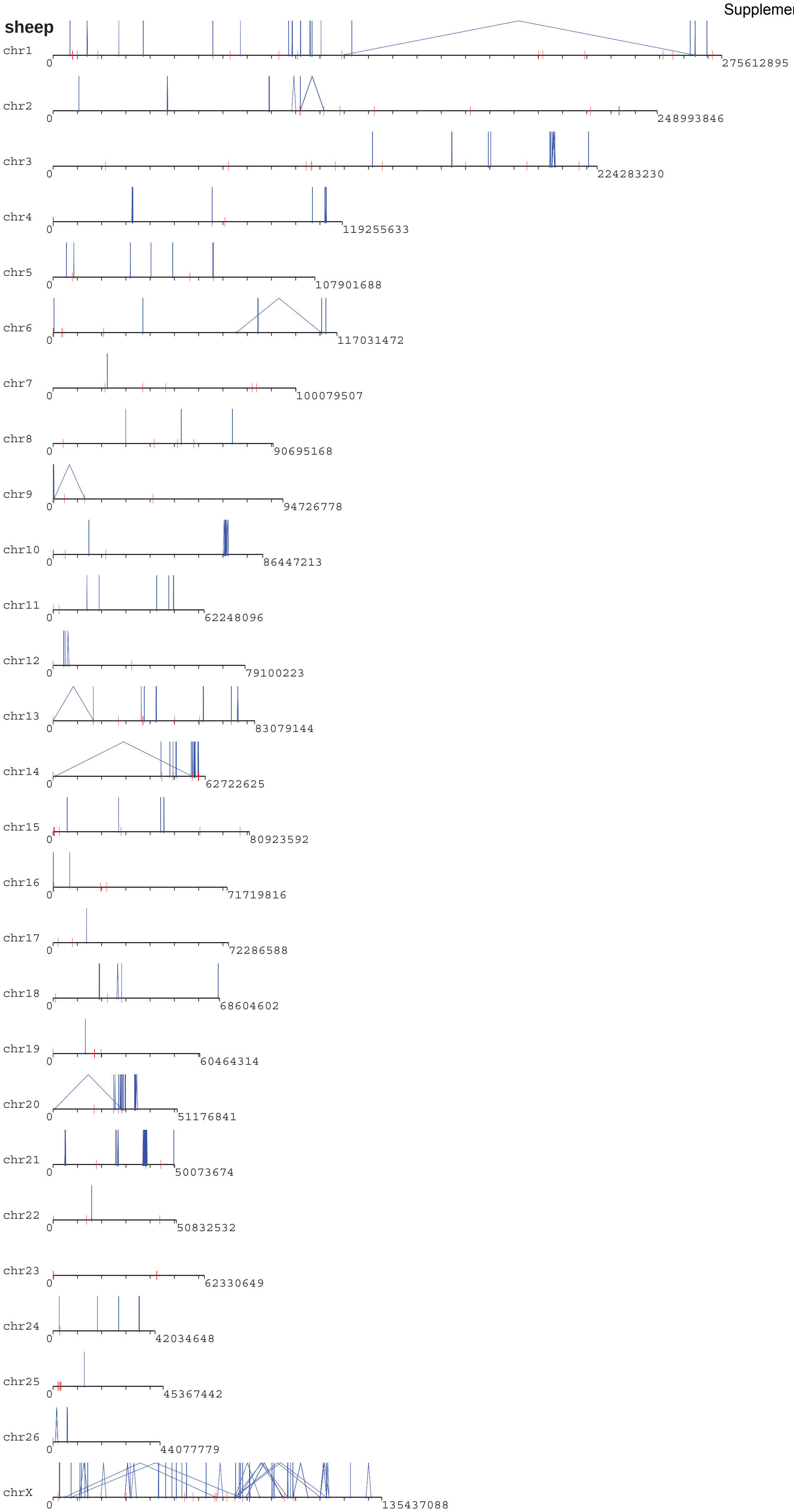
sheep
chr1
0
275612895
chr2
0
248993846
chr3
0
224283230
chr4
0
119255633
chr5
0
107901688
chr6
0
117031472
chr7
0
100079507
chr8
0
90695168
chr9
0
94726778
chr10
0
86447213
chr11
0
62248096
chr12
0
79100223
chr13
0
83079144
chr14
0
62722625
chr15
0
80923592
chr16
0
71719816
chr17
0
72286588
chr18
0
68604602
chr19
0
60464314
chr20
0
51176841
chr21
0
50073674
chr22
0
50832532
chr23
0
62330649
chr24
0
42034648
chr25
0
45367442
chr26
0
44077779
chrX
0
135437088

Supplemental Figure S1.7

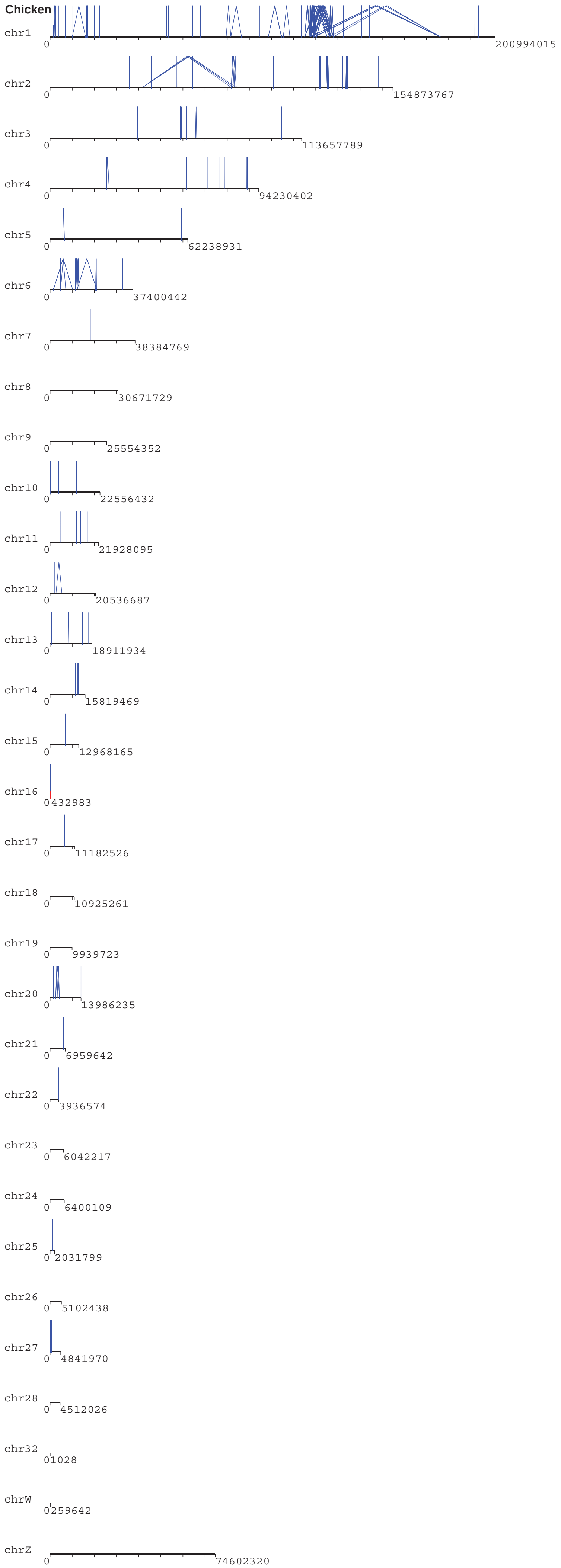

Supplemental Figure S2.1

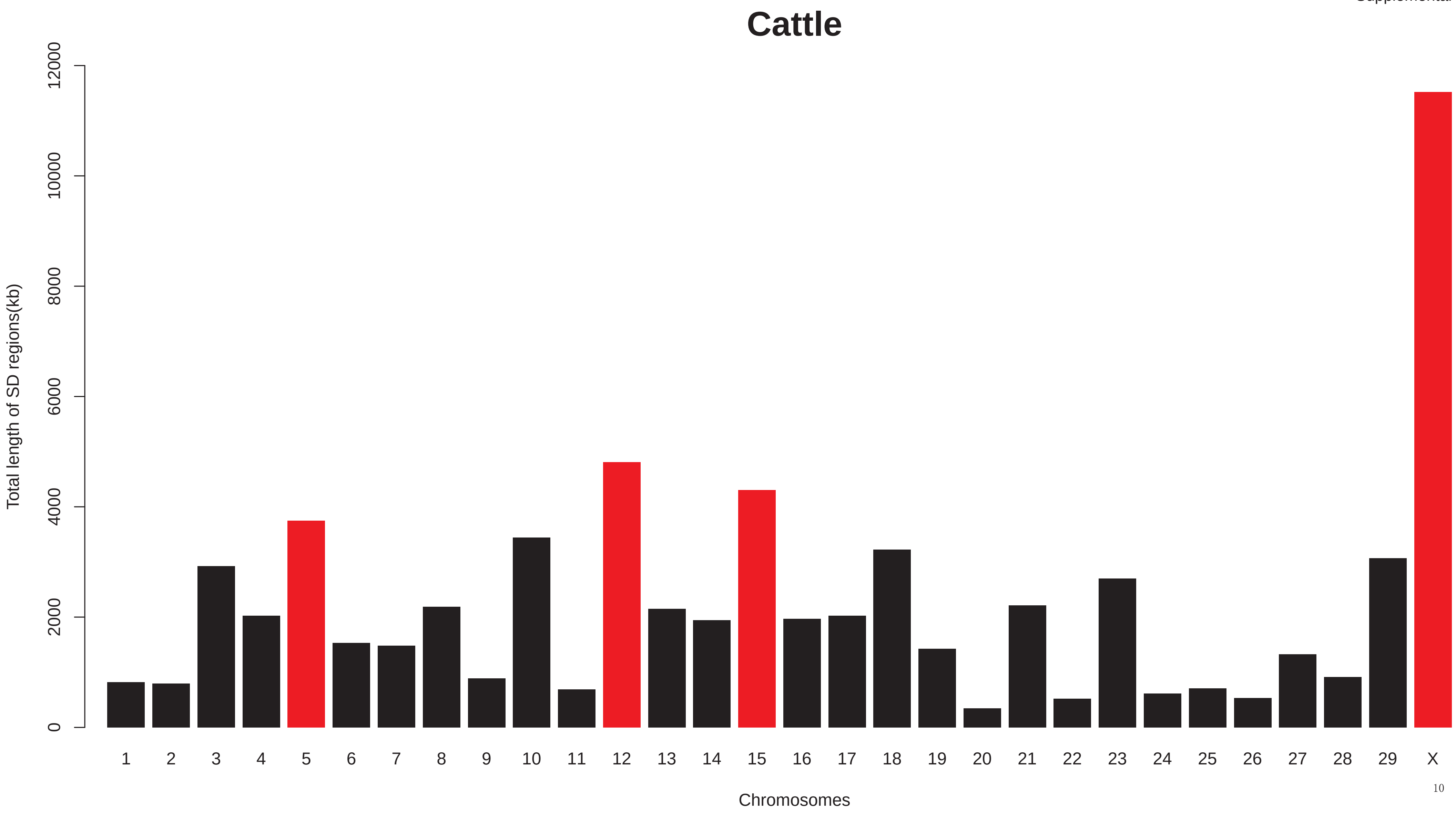

10

Supplemental Figure S2.2

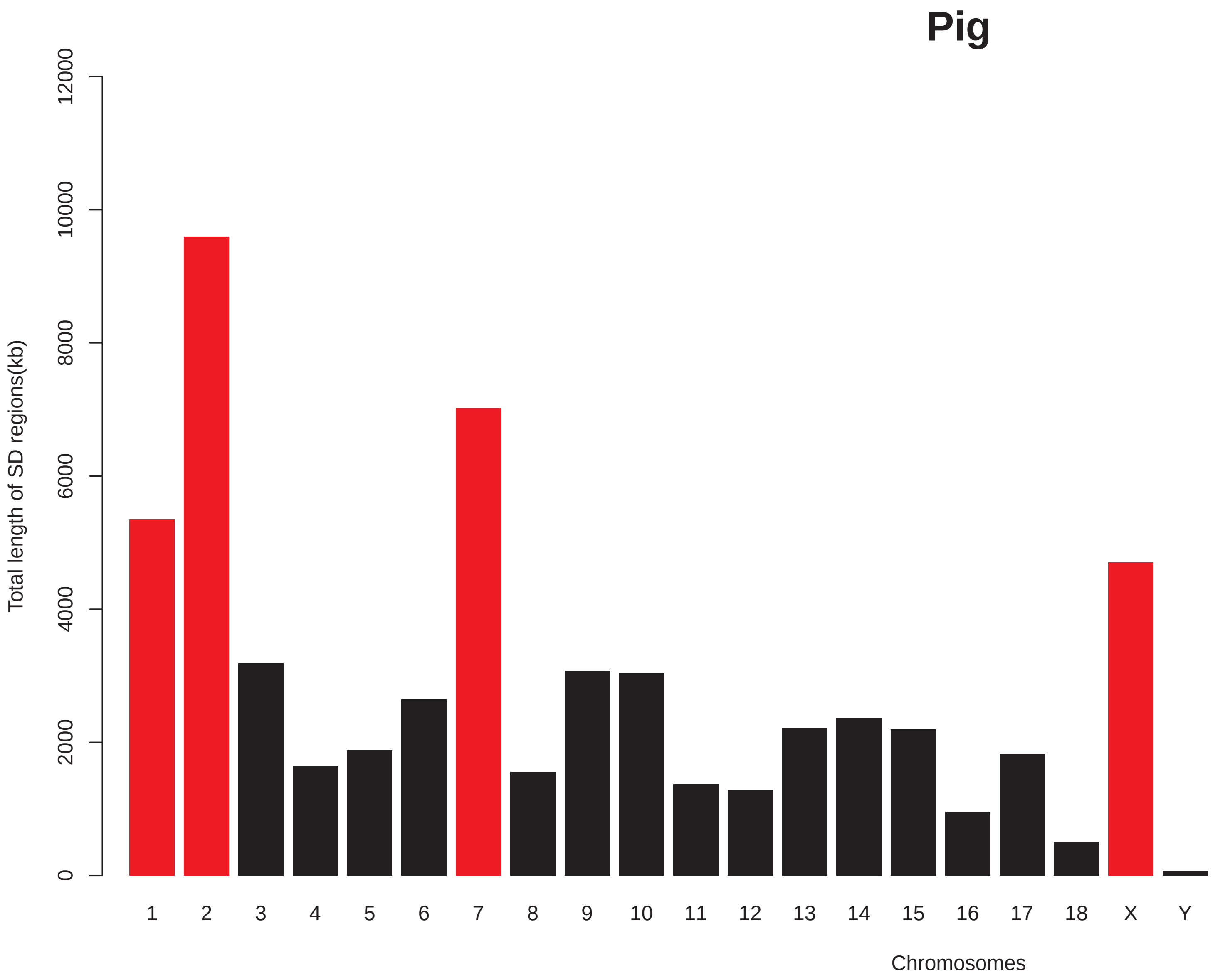

11

Supplemental Figure S2.3

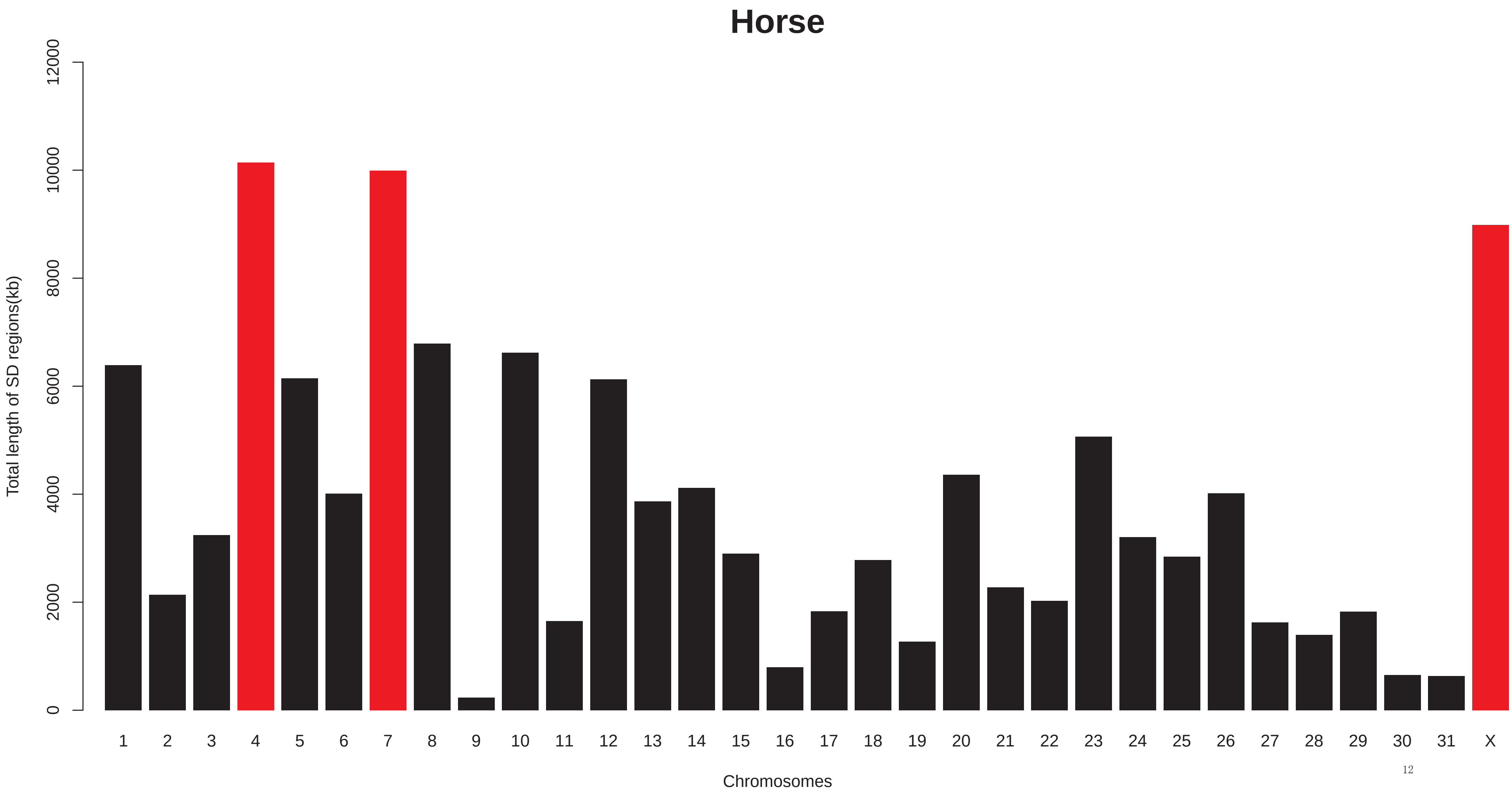

Supplemental Figure S2.4

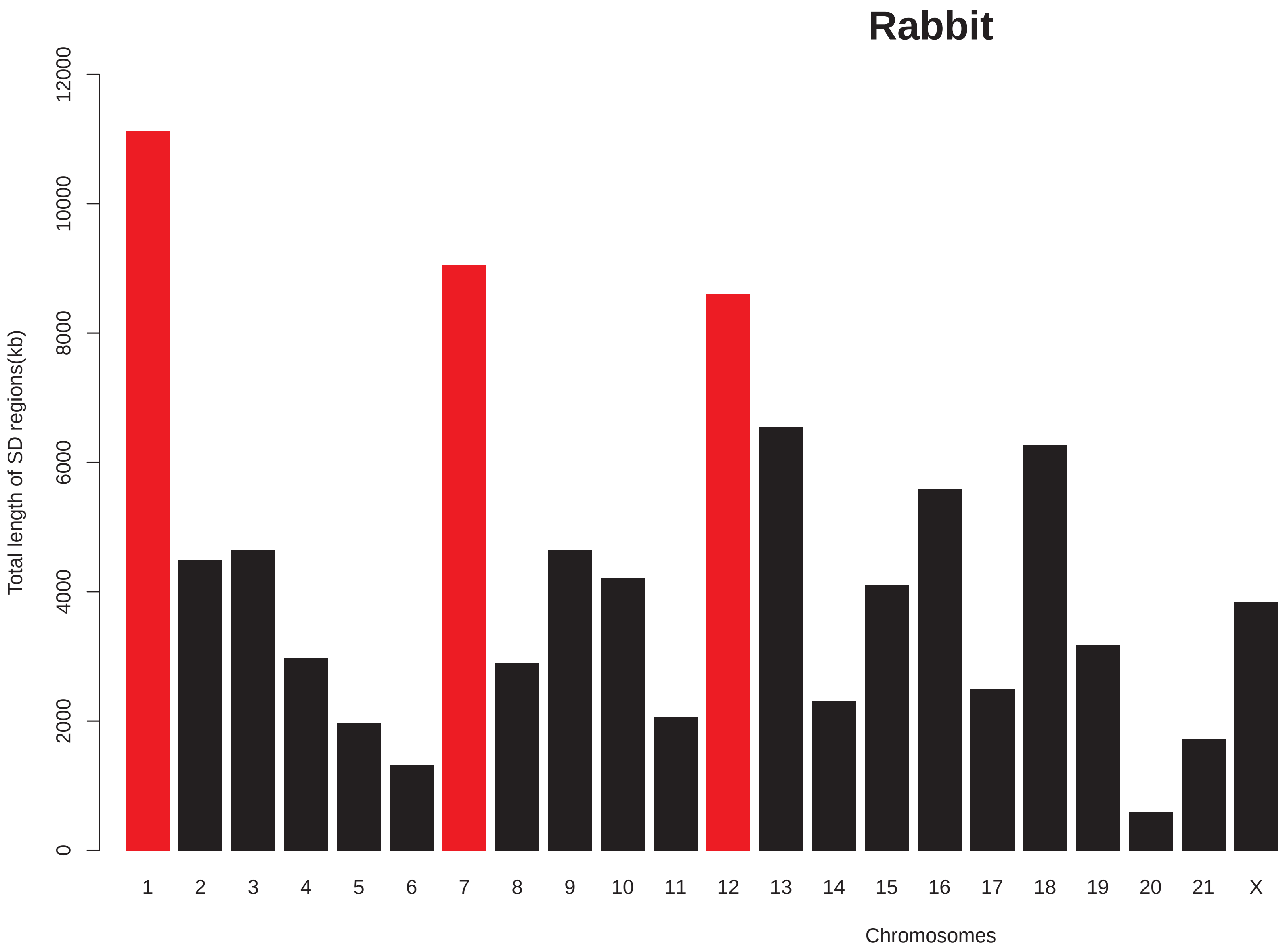

13

Supplemental Figure S2.5

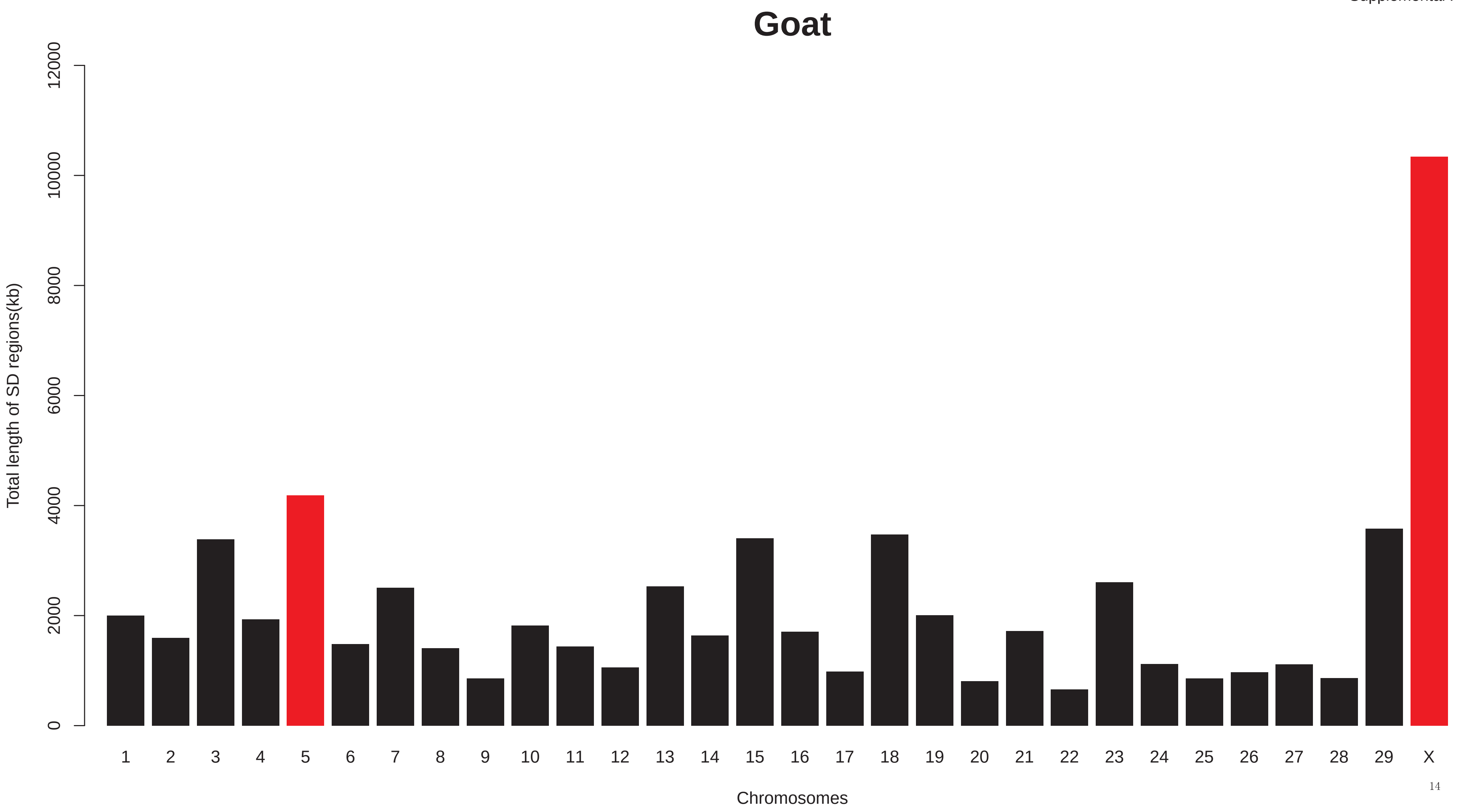

Supplemental Figure S2.6

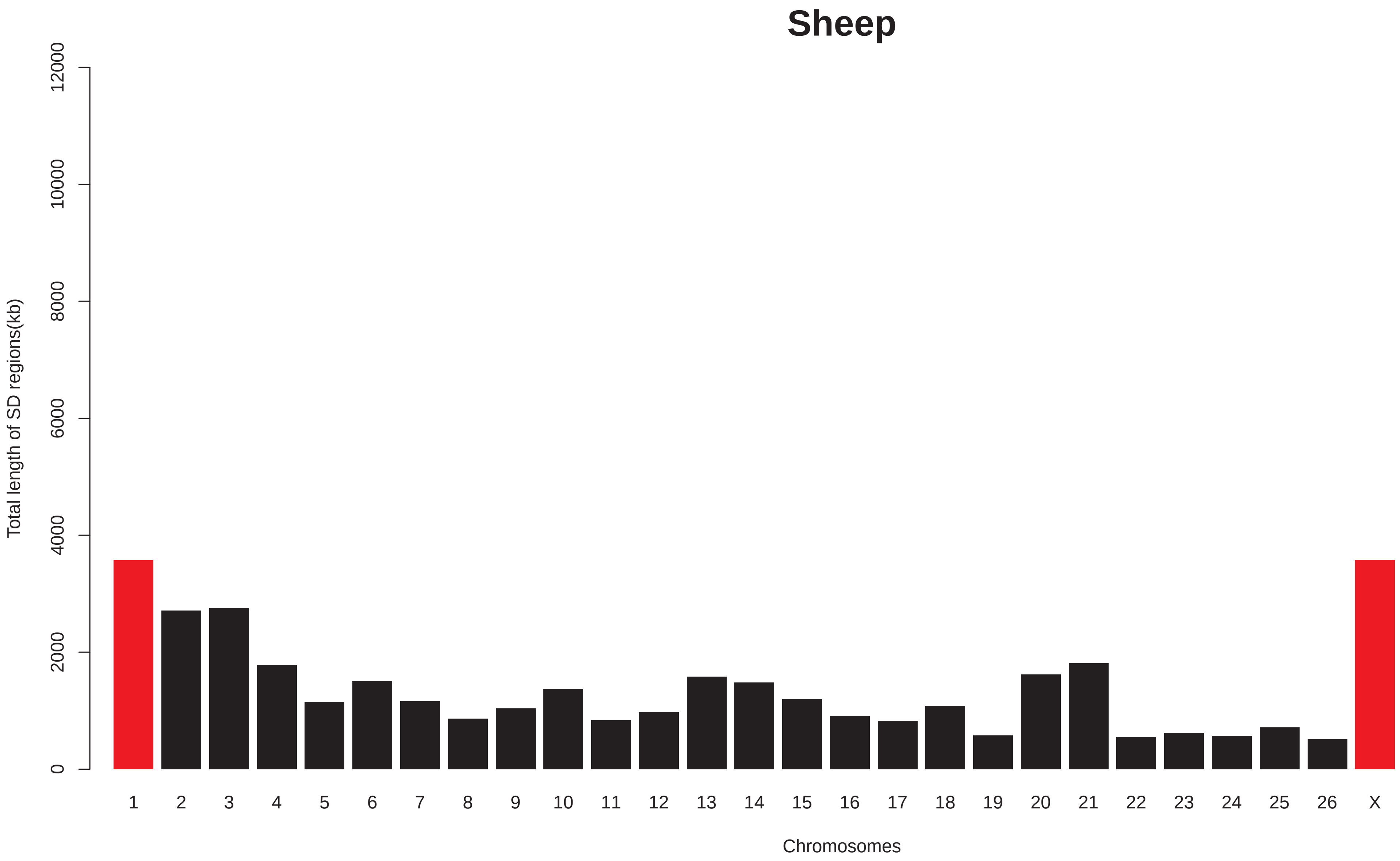

15

Supplemental Figure S2.7

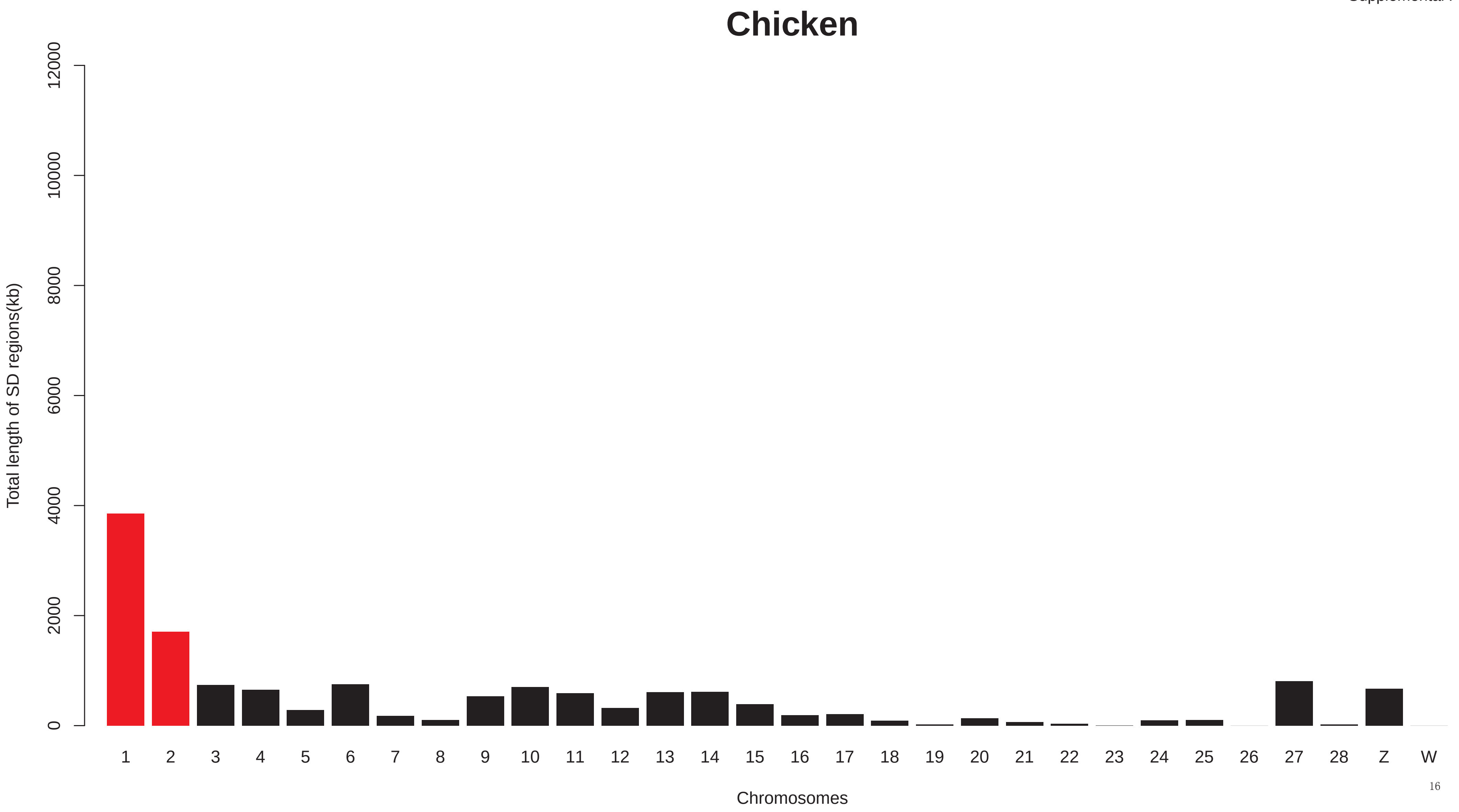

Cattle

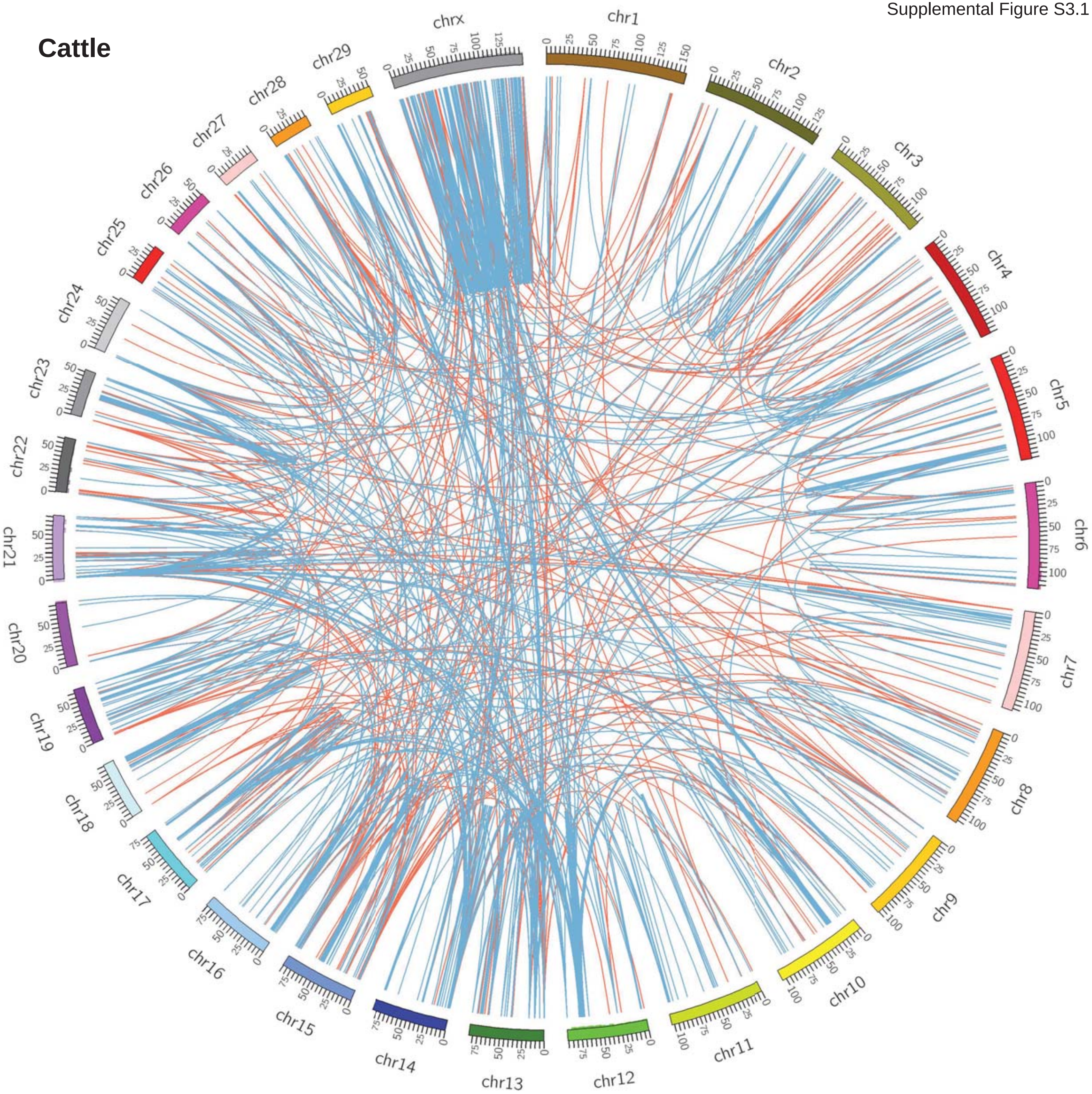
chrx
chr1
chr2
chr3
chr4
chr5
chr6
chr7
chr8
chr9
chr10
chr11
chr12
chr13
chr14
chr15
chr16
chr17
chr18
chr19
chr20
chr21
chr22
chr23
chr24
chr25
chr26
chr27
chr28
chr29

Pig

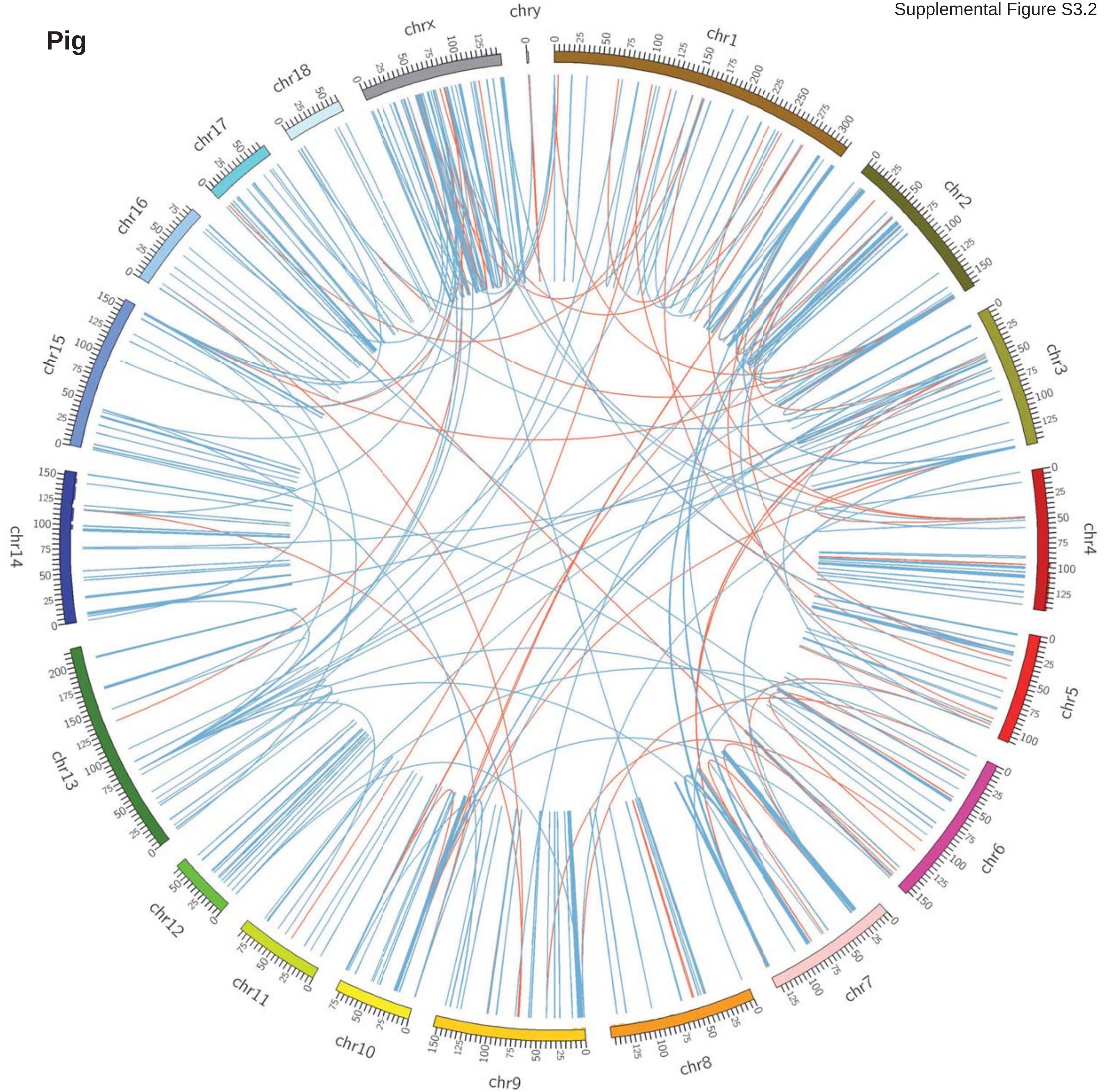

chry
chrx
chr1
chr2
chr3
chr4
chr5
chr6
chr7
chr8
chr9
chr10
chr11
chr12
chr13
chr14
chr15
chr16
chr17
chr18

Horse

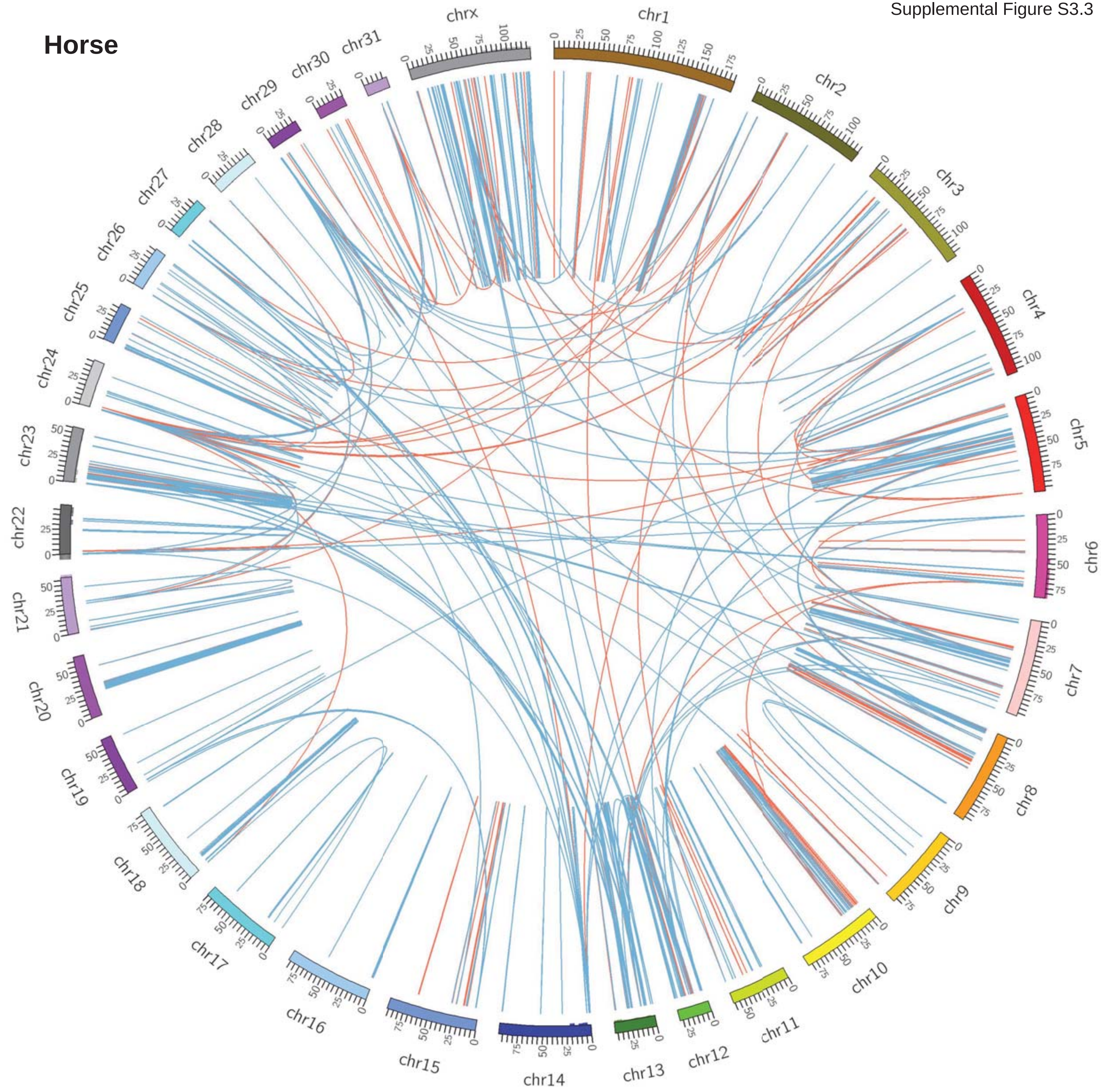
chrx
chr1
chr2
chr3
chr4
chr5
chr6
chr7
chr8
chr9
chr10
chr11
chr12
chr13
chr14
chr15
chr16
chr17
chr18
chr19
chr20
chr21
chr22
chr23
chr24
chr25
chr26
chr27
chr28
chr29
chr30
chr31

Rabbit

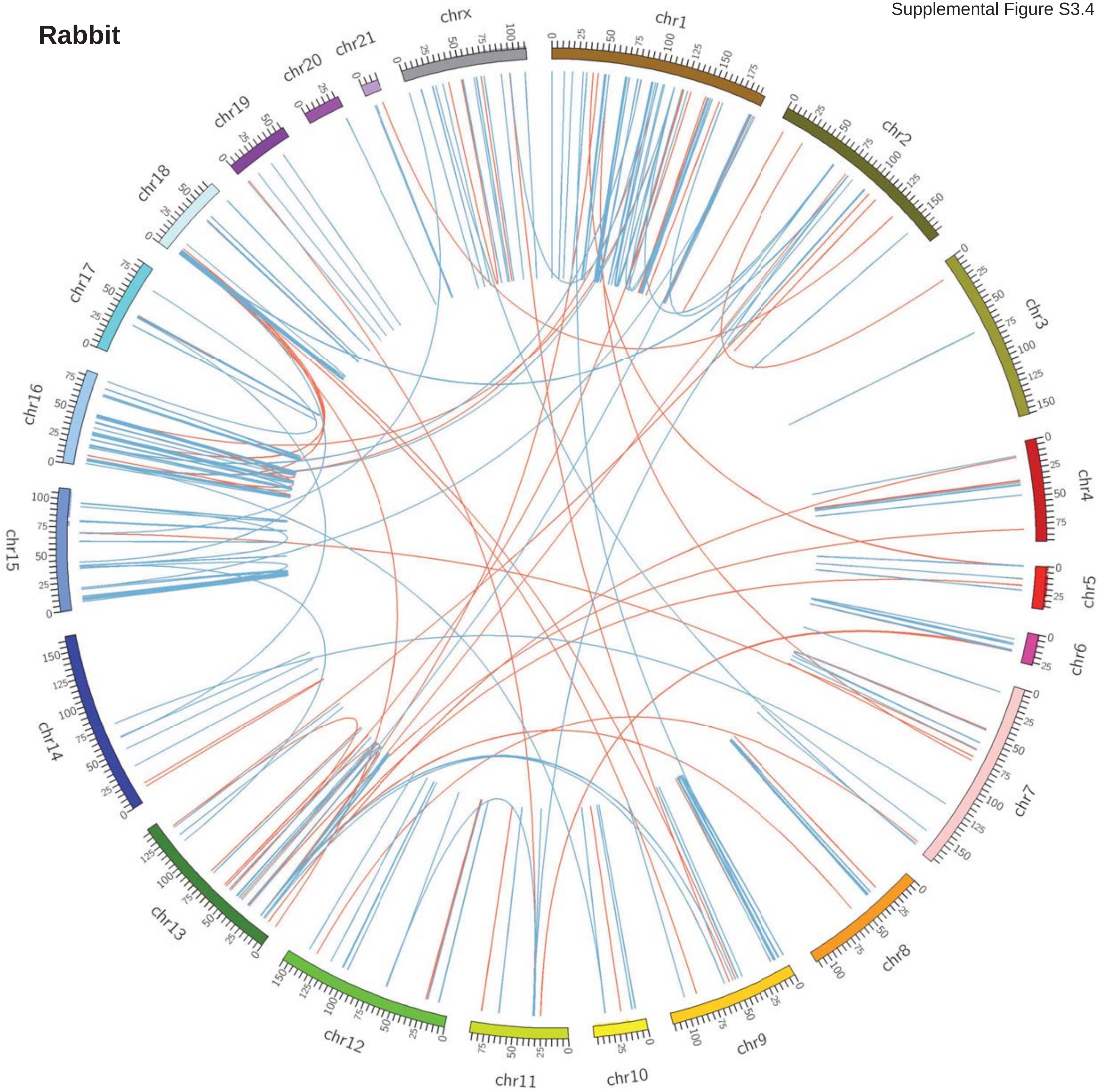
chrx
chr1
chr2
chr3
chr4
chr5
chr6
chr7
chr8
chr9
chr10
chr11
chr12
chr13
chr14
chr15
chr16
chr17
chr18
chr19
chr20
chr21

Goat

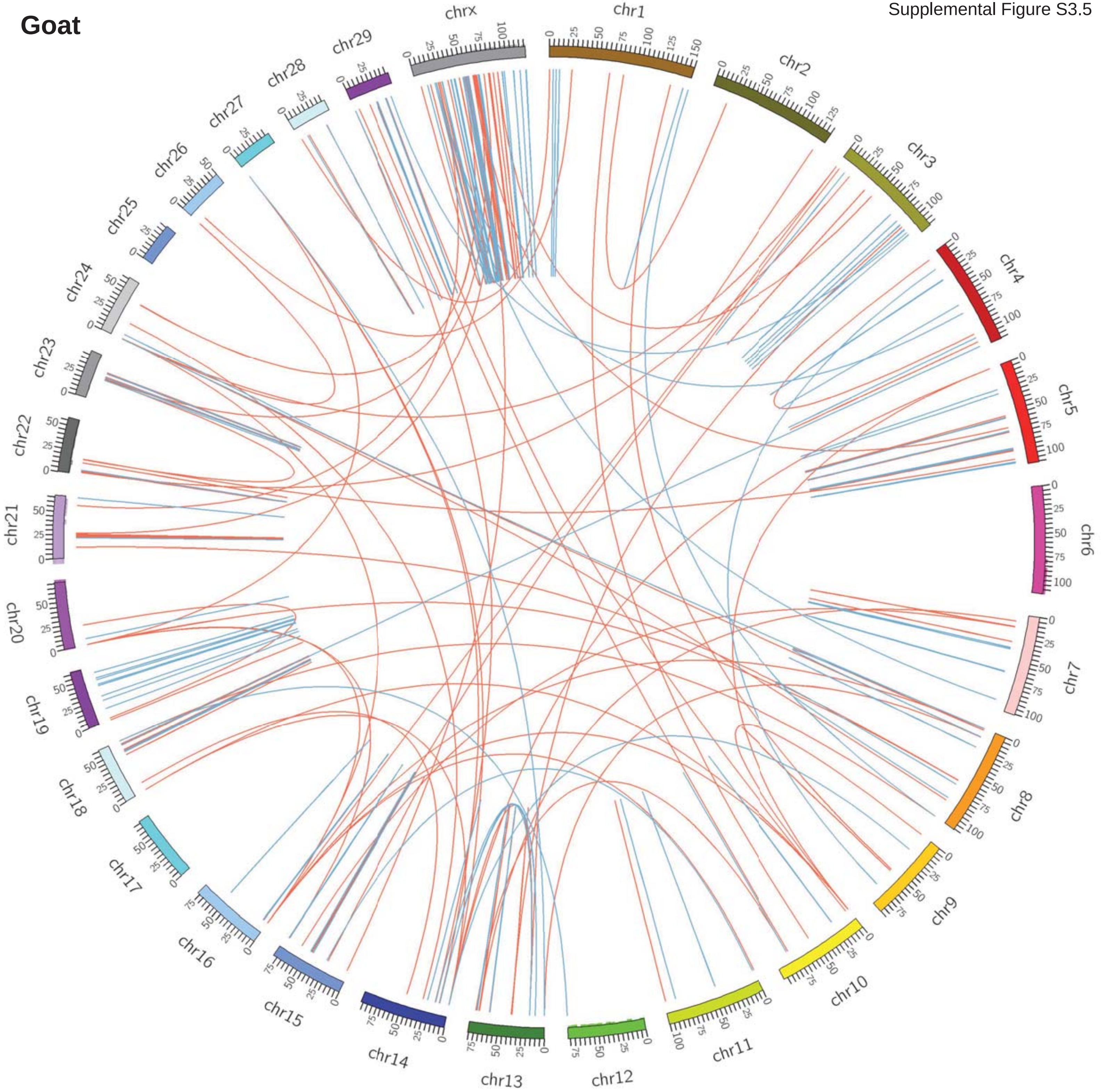
chrx
chr1
chr2
chr3
chr4
chr5
chr6
chr7
chr8
chr9
chr10
chr11
chr12
chr13
chr14
chr15
chr16
chr17
chr18
chr19
chr20
chr21
chr22
chr23
chr24
chr25
chr26
chr27
chr28
chr29

Supplemental Figure S3.6

Sheep

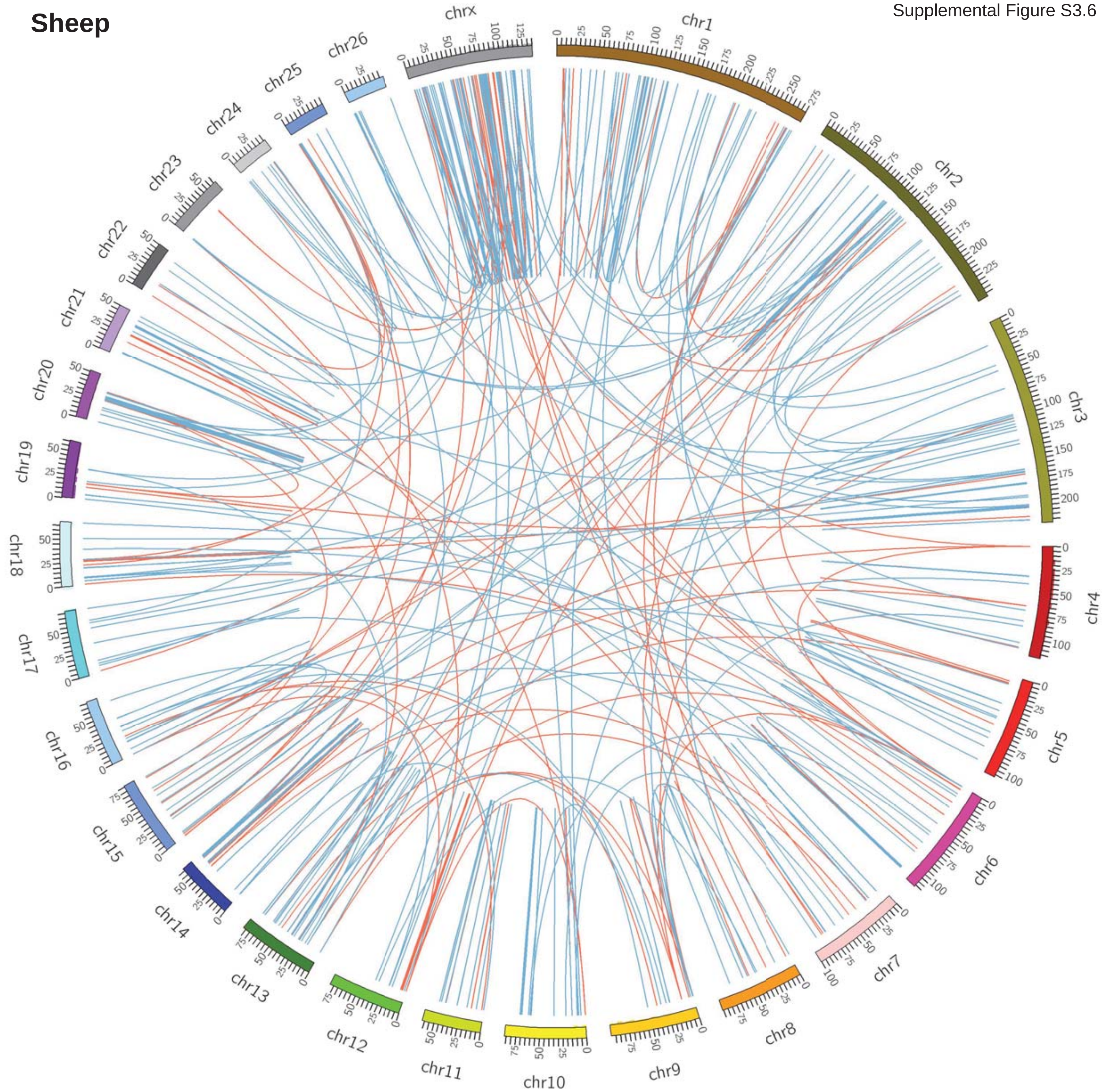

Chicken

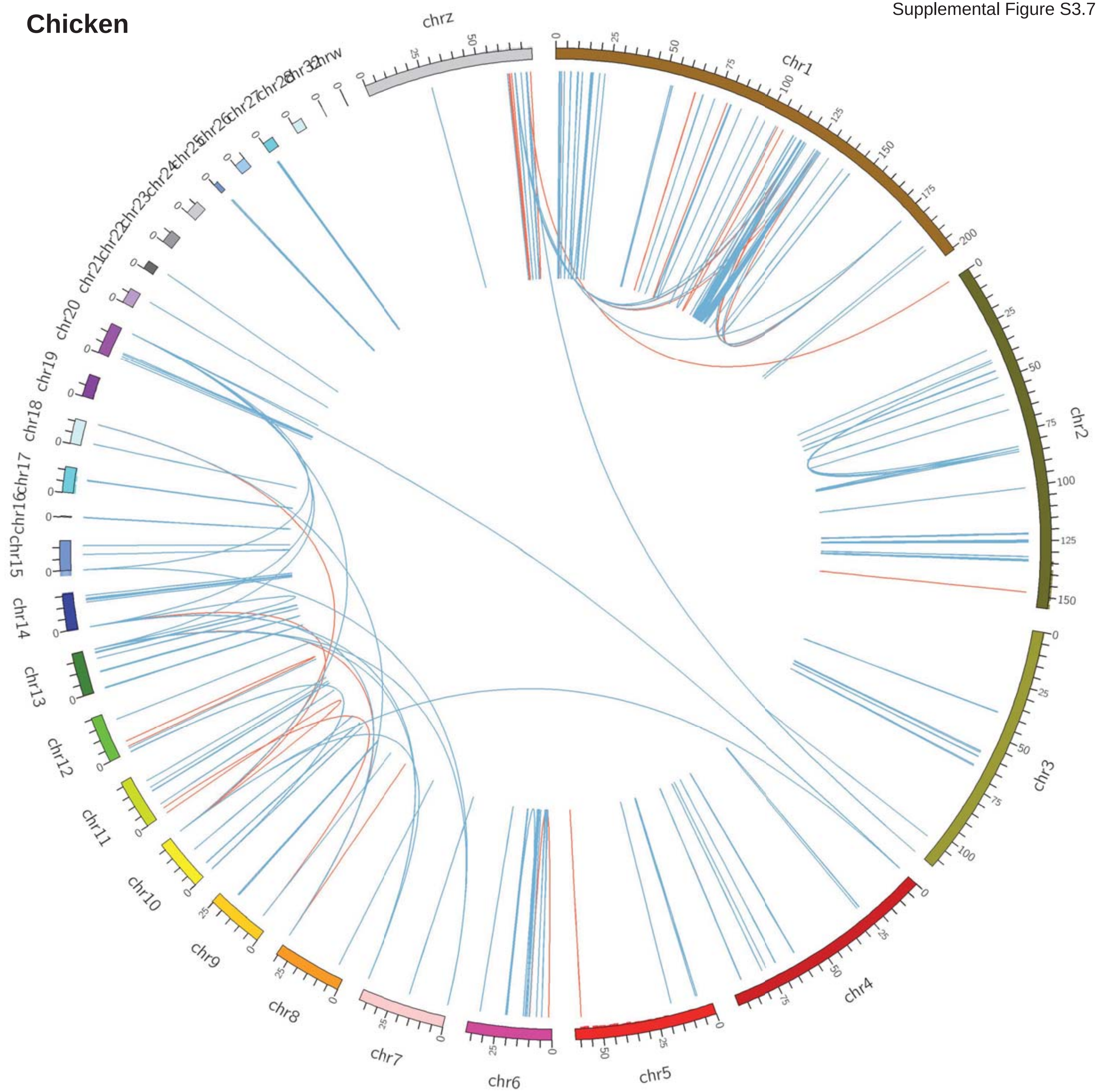
chrz
chr1
chr2
chr3
chr4
chr5
chr6
chr7
chr8
chr9
chr10
chr11
chr12
chr13
chr14
chr15
chr16
chr17
chr18
chr19
chr20
chr21
chr22
chr23
chr24
chr25
chr26
chr27
chr28
chr32
chrw
